# Single-cell multiplexed cytokine profiling of CD19 CAR-T cells reveals a diverse landscape of polyfunctional antigen-specific response

**DOI:** 10.1186/s40425-017-0293-7

**Published:** 2017-11-21

**Authors:** Qiong Xue, Emily Bettini, Patrick Paczkowski, Colin Ng, Alaina Kaiser, Timothy McConnell, Olja Kodrasi, Máire F. Quigley, James Heath, Rong Fan, Sean Mackay, Mark E. Dudley, Sadik H. Kassim, Jing Zhou

**Affiliations:** 1 Novartis Pharmaceuticals, 64 Sidney Street, Cambridge, MA 02139 USA; 2 IsoPlexis Corporation, 35 NE Industrial Rd, Branford, CT 06405 USA; 3 NanoSystems Biology Cancer Center, Division of Chemistry, California Institute of Technology, Pasadena, CA 91125 USA; 4 Department of Biomedical Engineering, Yale University, New Haven, CT 06520 USA; 5 Present Address: Novartis Institute of BioMedical Research, 300 Technology Square, Cambridge, MA 02139 USA; 6 Present Address: Novartis Institute of BioMedical Research, 64 Sidney street, Cambridge, MA 02139 USA; 7 Present Address: Novartis Pharmaceuticals, 45 Sidney Street, Cambridge, MA 02139 USA; 8 Present Address: Adaptimmune, 351 Rouse Blvd, Philadelphia, PA 19112 USA; 9 Present Address: Mustang Bio, 95 Sawyer Road, Waltham, MA 02453 USA

**Keywords:** Single-cell proteomics, CD19 CAR-T cell product, Polyfunctionality, Microfluidic microdevice, Precision profiling

## Abstract

**Background:**

It remains challenging to characterize the functional attributes of chimeric antigen receptor (CAR)-engineered T cell product targeting CD19 related to potency and immunotoxicity ex vivo, despite promising in vivo efficacy in patients with B cell malignancies.

**Methods:**

We employed a single-cell, 16-plex cytokine microfluidics device and new analysis techniques to evaluate the functional profile of CD19 CAR-T cells upon antigen-specific stimulation. CAR-T cells were manufactured from human PBMCs transfected with the lentivirus encoding the CD19-BB-z transgene and expanded with anti-CD3/anti-CD28 coated beads. The enriched CAR-T cells were stimulated with anti-CAR or control IgG beads, stained with anti-CD4 RPE and anti-CD8 Alexa Fluor 647 antibodies, and incubated for 16 h in a single-cell barcode chip (SCBC). Each SCBC contains ~12,000 microchambers, covered with a glass slide that was pre-patterned with a complete copy of a 16-plex antibody array. Protein secretions from single CAR-T cells were captured and subsequently analyzed using proprietary software and new visualization methods.

**Results:**

We demonstrate a new method for single-cell profiling of CD19 CAR-T pre-infusion products prepared from 4 healthy donors. CAR-T single cells exhibited a marked heterogeneity of cytokine secretions and polyfunctional (2+ cytokine) subsets specific to anti-CAR bead stimulation. The breadth of responses includes anti-tumor effector (Granzyme B, IFN-γ, MIP-1α, TNF-α), stimulatory (GM-CSF, IL-2, IL-8), regulatory (IL-4, IL-13, IL-22), and inflammatory (IL-6, IL-17A) functions. Furthermore, we developed two new bioinformatics tools for more effective polyfunctional subset visualization and comparison between donors.

**Conclusions:**

Single-cell, multiplexed, proteomic profiling of CD19 CAR-T product reveals a diverse landscape of immune effector response of CD19 CAR-T cells to antigen-specific challenge, providing a new platform for capturing CAR-T product data for correlative analysis. Additionally, such high dimensional data requires new visualization methods to further define precise polyfunctional response differences in these products. The presented biomarker capture and analysis system provides a more sensitive and comprehensive functional assessment of CAR-T pre-infusion products and may provide insights into the safety and efficacy of CAR-T cell therapy.

**Electronic supplementary material:**

The online version of this article (10.1186/s40425-017-0293-7) contains supplementary material, which is available to authorized users.

## Background

Adoptive immunotherapy is a novel treatment modality for human cancers [1, 2]. One such approach, CAR-T cell therapy, involves the use of viral vectors to genetically modify autologous T cells to express a chimeric antigen receptor (CAR) directed against a tumor antigen [2]. CD19 CAR-T, arguably the most successful CAR-T cell therapy to date in the clinic, involves genetically engineering autologous T cells ex vivo to express CARs against a B-lineage antigen CD19, which is expressed on tumor cells such as diffuse large B-cell lymphoma (DLBCL) and B-cell precursor acute lymphocytic leukemia (B-ALL) [3–5]. While striking results with a high rate of complete remission (67%–90%) have been observed in clinical trials in adults and children with relapsed/refractory B-ALL [3, 5–8], encouraging, yet variable, results have been reported with other B-cell malignancies treated by CD19 CAR-T therapy. An overall response rate of 57% (4 complete responders and 4 partial responders out of 14 patients) was reported in a small trial on relapsed/refractory chronic lymphocytic leukemia (CLL) and a complete response rate of 57% (4 out of 7 patients) was reported in a phase I study with chemotherapy-refractory DLBCL [9]. These variable clinical responses across patients may result from many factors such as pre-conditioning regimen of patients, CAR-T cell administration procedure as well as T cell sources and manufacturing process. However, the key contributors to the variations are not fully identified [10, 11]. High-resolution single-cell analysis of functional attributes of CAR-T pre-infusion products may provide new insights into the variability of clinical responses and open up new avenues for the development of more efficacious yet safe CAR-T cell therapy.

In an in vitro system, the reorganization of tumor antigen by CAR, are followed by a wave of signals that comprise the secretion of cytolytic enzymes, stimulatory cytokines, and chemoattractive proteins. How these signals reflect CAR-T function in patient is minimally characterized and poorly understood. This is further compounded by the secretion of immunosuppressive cytokines that reduce the efficacy of treatment or pro-inflammatory cytokines involved in immunotoxic side effects such as cytokine release syndrome (CRS) [12]. Moreover, this signal, which varies between patients and differs in individual CAR-T cells, is difficult to control or standardize, highlighting a pressing need to systematical evaluate the ability of engineered CAR-T cell product to release these signals following an antigen-specific challenge and to link this capability to clinical outcomes.

It is well accepted that T cells capable of co-producing multiple cytokines/chemokines at the single cell level, termed “polyfunctional” T cells, are the key effector cells contributing to the development of potent and durable cellular immunity against viral infection or cancer [13–16]. The same principle is expected to apply to engineered CAR-T cells in autologous cellular immunotherapy. Porter et al. showed that CD19 CAR-T-cells collected from CLL patients following treatment are indeed polyfunctional measured by 6-color ICS-FC [9]. Four patients exhibited complete remissions, and, for those patients, their CAR-T cells persist and retain polyfunctionality for up to 4 years post infusion [9]. This study highlighted the importance of polyfunctionality, for therapeutic efficacy of anti-tumor CAR-T cells. While most studies characterized single-cell cytokine production in CD19 CAR-T cells using intracellular staining flow cytometry (ICS-FC) [17], the limited number of cytokine/chemokines measured per single cell undermines the capability to comprehensively assess CAR-T polyfunctionality.

A comprehensive assessment of CAR-T polyfunctionality has become possible with the development of a single-cell barcode chip (SCBC) microdevice that demonstrated simultaneous measurement of up to 42 cytokines secreted from single cells and thousands of single cells analyzed in parallel per device [18]. In the current study, we apply this microdevice technology to CD19 CAR-T cells to delineate the landscape of effector function capability in response to antigen-specific stimulation. We were able to load small quantities of CAR+ T cells onto the device and distinguish CD4 and CD8 cells using high content imaging cytometry. We then simultaneously measured 16 cytokines/chemokines from thousands of single CAR-T cells. Elevated cytokine/chemokine levels in response to antigen specific stimulation at the single-cell level were observed for cells generated from all 4 donors, albeit with significant donor-to-donor variation. Significant subsets of stimulated CAR-T cells exhibit high polyfunctionality with a dominant anti-tumor effector cytokine profile. New bioinformatics tools were developed to visualize polyfunctional T cell subsets and compare across donors. The analysis revealed distinct functional clusters from each donor and provided a landscape of CD19 CAR-T cell effector functions upon antigen stimulation. The comprehensive single CAR-T cell analysis paves the way to understand the relationship between in vitro function profiles and therapeutic outcomes.

## Methods

### Fabrication of antibody barcode slides

The silicon master for antibody barcode manufacturing, polydimethyl siloxane (PDMS) was etched with the deep-reactive-ion etching (DRIE) method. It was pretreated with trimethylchlorosilane (Sigma-Aldrich) vapor in a vacuum desiccator for 30 min. The master was then compressed into an acrylic base, to form the mold for PDMS chips. The PDMS pre-polymer elastomer base and curing agent, Sylgard 184 (Dow Corning) was mixed completely (parts A and B in a 10:1 ratio) and placed in a vacuum desiccator for 30 min to remove air bubbles. The mixture was then injected into the mold using a syringe and the mold was cured in the oven at 80 °C for 1.5 h. After curing, the PDMS layer was removed from the mold and holes for the inlet and outlet ports were punched. Each flow-patterning PDMS chip measured 75 mm (length) × 25 mm (width) × 6 mm (height). The PDMS microchip contained 20 separate microchannels (20 μM width × 20 μM depth) arranged in a serpentine pattern across the chip that can pattern up to 20 different solutions respectively. The PDMS microchip was then bound to a poly-L-lysine glass slide to form an antibody flow pattern device. 2 μL of different capture antibodies (Additional file 1) were flowed into each microchannel through the PDMS chip for 3 h. The antibodies were immobilized on the glass slide by binding to the poly-L-lysine. After flow patterning, the PDMS was removed from the slide and the antibody barcode slide was blocked for 30 min in 3% BSA/PBS. The slide was then washed sequentially in PBS (Lonza), 50% PBS/DI water, DI water, DI water and dried for 30 s in a Labnet slide spinner (C1303-T). The slide was vacuum sealed and stored indefinitely at −20 °C prior to use.

### Fabrication of microchamber array chips

The PDMS microchamber array chip was prepared using the microchamber array silicon master following the same steps as described above. The PDMS microchamber array chips were 25 mm (width) × 55 mm (length) × 4.75 mm (height). Each array contained 24 columns of 530 microchambers per column (totaling 12,720 microchambers per chip). Each microchamber measured 20 μM (width) × 2060 μM (length) × 20 μM (depth) for a total volume of ~1.2 nL per chamber.

### Generation of healthy CD8 T cells

Healthy PBMCs were isolated from whole blood (StemCell) using Ficoll-Paque Plus (GE Healthcare) density gradient centrifugation at 300 g for 20 min. The interphase containing PBMC was harvested and stimulated in complete X-VIVO 15 media (Lonza) at a cell density of 1 × 10^7^/mL with immobilized anti-CD3 antibody (10 μg/mL, eBioscience) and soluble anti CD28 antibody (4 μg/mL, eBioscience) at 37 °C, 5% CO_2_ for 48 h. The CD8 T cells then were enriched by magnetic bead-conjugated anti-CD8 antibody (Miltenyi-Biotec) prior to SCBC assay and ICS assay.

### ICS assay of healthy CD8 T cells

The enriched CD8 T cells were stimulated with PMA (50 ng/mL, Sigma-Aldrich) and Ionomycin (1 μg/mL, Sigma-Aldrich) at a density of 1 × 10^6^/mL in fresh complete X-VIVO 15 media at 37 °C, 5% CO_2_ for 6 h with brefeldin A (1 μg/mL, eBioscience) the last 3 h. Cells were then fixed with 4% paraformaldehyde (Sigma-Aldrich), permeabilized with 0.5% saponin (Sigma-Aldrich), and stained with PE-conjugated anti-IFN-γ /PE-CY7-conjugated anti-TNF-α. The cells were acquired by S3e cell sorter (BioRad) and analyzed by Flowjo software v10 (TreeStar Inc.).

### Generation of CD19 CAR-T cells

The CD19-BB-z transgene lentiviral vector was designed and produced as described before [3, 19, 20]. Apheresis of healthy donors (2 females and 2 males with age range 26–51) was obtained from HemaCare. Methods of CAR-T cell preparation have been previously described [20–23]. Briefly, peripheral blood mononuclear cells (PBMCs) were isolated from the apheresis and activated with anti-CD3/anti-CD28 monoclonal antibody-coated magnetic beads (Thermo Fisher) in a modified X-VIVO 15 media. Cells were transduced with a lentiviral vector encoding the CD19-BB-z transgene and ex vivo expanded in a closed system for 10 days. Cell concentration was monitored during the expansion process and fresh medium was added every 2–3 days. On the day of harvest, transduced CAR-T cells were isolated from the population using anti-PE microbeads (Miltenyi) following the manufactures’ instruction. Briefly, cells were incubated with a PE conjugated antibody specific for CARs on cell surface, followed by a second incubation with anti-PE microbeads. After wash, the cell-beads mixture was passed through a LS (Miltenyi) column in a MACS separator. Following another wash step, the CAR positive fraction was collected by plunging out the column-retaining cells and cryopreserved. The collected cells were analyzed using flow cytometry to confirm that >90% of the cells were CAR+. Before any assay, cryopreserved CAR-T cells were thawed and cultured overnight at a concentration of 1 × 10^6^ cells/mL in X-VIVO 15 media. Immediately prior to use, dead cells were removed from culture using a Dead Cell Removal Kit (Miltenyi) and LS Magnetic Column (Miltenyi).

### Conjugation of Dynabeads for stimulation

The tosylactivated M-450 Dynabeads (Invitrogen) were conjugated with a CAR specific antibody following the manufacturers’ instructions. In short, 4E8 beads were incubated with 200 μg antibodies for 24 h. After extensive wash, the beads were aliquoted and stored at 4 °C until use. Beads conjugated with non-specific IgG were generated as the same time and used as negative control.

### CAR-T cell population assays

CD19 CAR-T cells (1 × 10^6^ cells/mL) were mixed at a 1:4 ratio with beads and cultured in wells of a 96 well tissue culture plate for 24 h. The supernatant was harvested and analyzed using a human IFN-γ quantikine ELISA kit (R&D) or a multiplexing population assay developed in house. The ELISA assay was performed following manufacturer’s instructions. Briefly, the assay plate was incubated with samples, antibody conjugate, substrate solution and stop solutions in sequential order. The plate was read using a SpectraMax M5 plate reader (Molecular Device). The multiplexing population assay was performed using a custom PDMS device. A custom 12-hole PDMS microwell slab (each hole at D = 1 cm) was placed on top of a 16-plex antibody barcode slide to form a multiplexing 12-well assay device. The two components were tightly clamped with screws using a custom polycarbonate plate clamping system. 50 μL of cell culture supernatant was added to each well and incubated for 1 h. Following incubation, the wells were washed 3 times with 1% BSA/PBS and then incubated with 50 μL of detection antibody cocktail (1:200 dilution in 1% BSA/PBS, Additional file 1) for 1 h. The wells were then washed again 3 times with 1% BSA/PBS and incubated with 50 μL of APC streptavidin (1:100 dilution in 1% BSA/PBS, BioLegend) solution for 30 min. After washing 5 times with 1% BSA/PBS, the slide was removed from the clamp. The slide was washed in a Coplin jar for 3 min at 125 rpm sequentially using PBS, 50% PBS/DI water, DI water, DI water sequentially. The slide was then dried for 30 s in a Labnet slide spinner (C1303-T), scanned with a GenePix 4400A microarray scanner and analyzed using IsoPlexis’ proprietary IsoSpeak software.

### Preparation of cell suspension for SCBC assay

The healthy CD8 T cells from healthy PBMCs were suspended at a density of 1.25 × 10^6^/mL in fresh complete X-VIVO 15 media with the addition of PMA (50 ng/mL) and Ionomycin (1 μg/mL). CD19 CAR-T cells (1 × 10^6^ cells/mL) were mixed with beads at a 1:4 ratio. The mixture was plated into a single well of a 96-well plate and incubated at 37 °C for 6 h. Stimulated cells were then collected and incubated with anti-human CD4 RPE antibody (1:100 dilution, Thermo Fisher) and anti-human CD8a Alexa Fluor 647 antibody (1:100 dilution, BioLegend) at room temperature for 10 min. The cells were then spun at 300 g for 10 min and re-suspended in fresh media at a density of 1.25 × 10^6^ cells/mL.

### SCBC assay

The PDMS microchamber array was plasma treated for 2.5 min with a Plasma Etch PE-25 plasma cleaner and blocked in 3% BSA/PBS for 30 min. Immediately before the assay, the PDMS chamber was rinsed with media and blown dry using compressed air. It was then placed on a glass slide and secured into a custom clamping system. 30 μL of cell suspension was pipetted onto the microchamber array chip. The antibody barcode slide was put on top of the microchamber PDMS with the antibody side facing down. The whole system was clamped tightly in a custom clamping system. The system was imaged immediately as described in **Microchamber array imaging.** Post imaging, the microchamber assembly was placed in a standard 5% CO_2_, incubator at 37 °C for 16 h. Following incubation, the microchamber system was dissembled in a 1% BSA/PBS bath and the antibody slide was removed and rinsed with 1% BSA/PBS. The slide was then incubated with 300 μL detection antibody cocktail (Additional file 1) for 45 min. Each detection antibody is at a concentration of 0.25 μg/mL in 1% BSA/PBS solution. Following this step, the barcode slide was rinsed with 1% BSA/PBS and incubated with 300 μL APC streptavidin solution (1:100 dilution in 1% BSA/PBS, BioLegend) for 30 min. The slide was rinsed with 1% BSA/PBS again and then washed in Coplin jars sequentially using PBS, 50% PBS/DI water, DI water, DI water for 3 min at 125 rpm. The slides were dried for 30 s in a Labnet slide spinner (C1303-T) and scanned using a GenePix microarray scanner as described in **Antibody barcode slide imaging**.

### Microchamber array imaging

The assembled microchamber system was imaged using a Zeiss Axio Observer.Z1 fluorescent microscope with a Hamamatsu Orca-Flash4.0 LT Digital CMOS camera (C11440-42 U) and an automatic stage. For each microchamber array, bright field images were taken to visualize the microchambers and fluorescent images were taken to visualize cells labeled with CD4 or CD8 antibodies. A set of 253 tiles (5X image) or 494 tiles (10X image) was taken for the entire array (54183uM x 24866uM). Using Zen2 Pro software, the tiles were exported as TIFF image files for analysis.

### Antibody barcode slide imaging

A GenePix 4400A scanner (Molecular Devices) was used to scan the antibody barcode slides. Each slide was scanned using a GenePix Pro software (Molecular Devices) at two color channels, 488 (blue, PMT 350, Power 90) and 635 (red, PMT 600, Power 90). The image was then exported as a TIFF file for analysis.

### Image processing and data analysis

All data extraction and visualizations were performed using IsoPlexis’ IsoSpeak software package. The application was developed using C++ and the QT application framework, and used the open source OpenCV library for image processing. The microchamber array images and antibody barcode images were overlaid based on visual alignment markers embedded in the SCBC. Cell counts and phenotypes in each microchamber could therefore be mapped to the corresponding cytokine/chemokine levels. The software automatically located cell chambers using edge and contour detection. The software identified and counted cells in each microchamber based on fluorescence intensities in the fluorescence images and feature detection in the brightfield image. Cell subsets (CD4/CD8) were further determined based on immunofluorescence. User verification was performed on a small subset (normally around 2–3% of the whole array) to validate the automatic results. The software analyzed the antibody barcode image to determine the location of each antibody lane, using Hough transforms and subsequent interpolation. The raw intensity value of each cytokine readout from each single-cell was extracted from the image region where the single cell microchamber overlaps the cytokine’s corresponding antibody lane. Zero-cell microchambers and their associated protein signals were used to assess cytokine-specific background. A background threshold was computed per cytokine, defined as three standard deviations above the average zero-cell signal intensity. Signals with a signal-to-noise ratio (SNR) of at least 2 (relative to the background threshold) were considered as true protein signals. If at least 20 single cells or 2% of all single cells (whichever quantity was larger) showed true protein signal for one specific cytokine, this cytokine was labeled as significantly secreted by the sample. Knowing the secretion profile of each single-cell, the frequency of all polyfunctional groups present in the sample were identified. From these frequencies and the background-subtracted signal intensities, the polyfunctional strength of the sample, and the percent contribution from each cytokine group was found. PAT PCA transformations were performed as outlined in Additional file 2: Figure S3. All visualizations were automatically generated in IsoSpeak from the above data.

## Results

### Study design - highly multiplexed measurement of cytokine secretions from single CD19 CAR-T cells upon antigen-specific stimulation

**Fig. 1:**
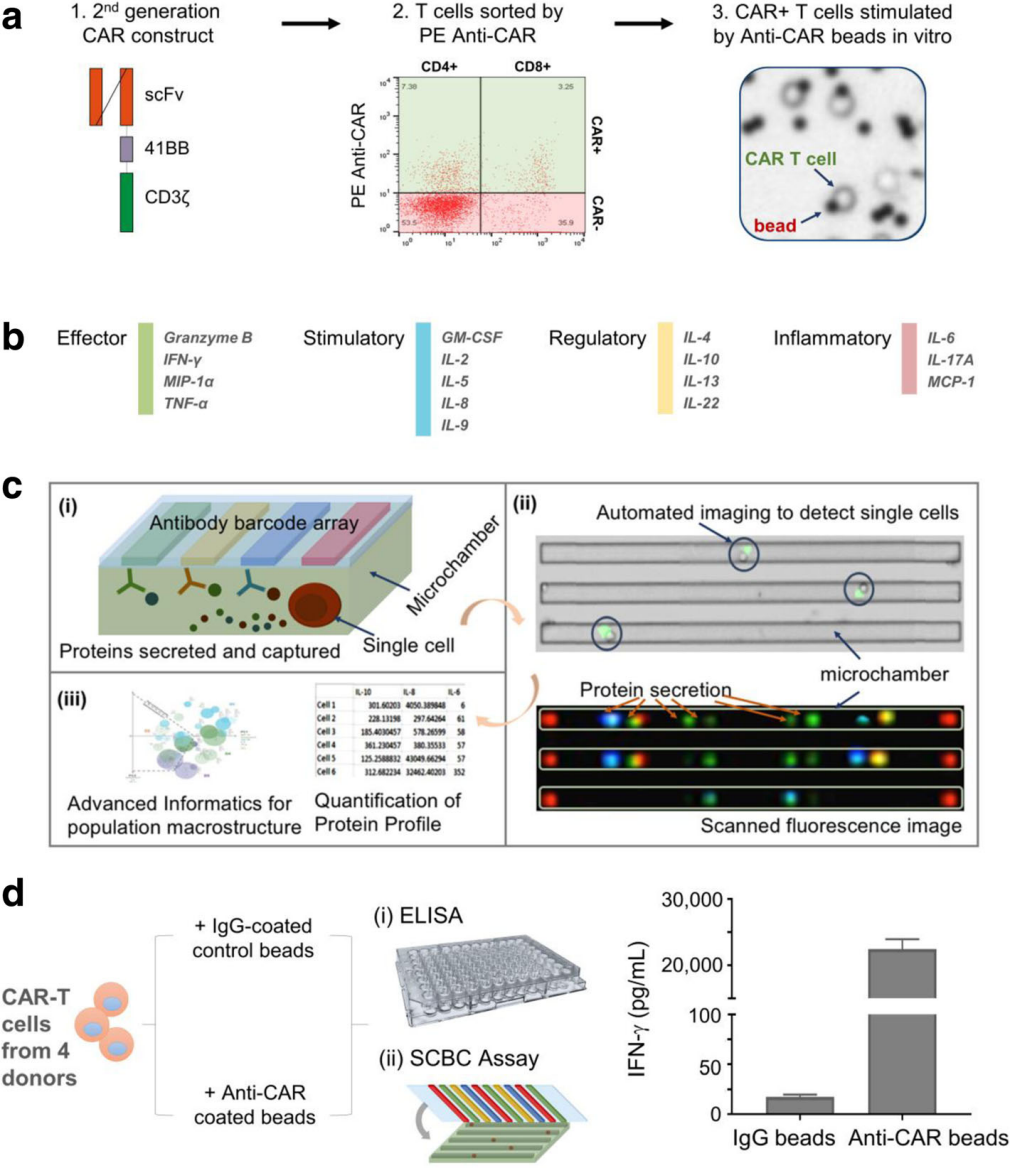
Multiplexing single cell measurement of CAR-T cells in microchambers. **a** Schematic outline of CD19TL19 CAR-T cell generation, sorting and stimulation. The CD19-BB-z transgene lentiviral vector was used for CAR-T cell generation. CAR-T cells were sorted and then stimulated by anti-CAR beads prior to the SCBC assay. **b** The validated 16-plex panel including 4 groups of cytokines: effector, stimulatory, regulator and inflammatory. **c** Major work flow for single-cell functional proteomic analysis. (i) Schematic depiction of a microchamber fabricated in PDMS used for isolating single cells and assaying a panel of proteins/cytokines secreted from the entrapped cell with an antibody barcode array patterned on the glass slide. Each device has 12,000 microchambers. (ii) Motorized miscopy allows for automated imaging of the entire microchamber PDMS device for locating and counting T cells in microchambers. Protein secretion profile is obtained by quantifying the fluorescence signals corresponding to single-cell secretions in each microchamber. Overlay of these two data sets allows for the identification of single-cell protein secretion profiles. (iii) Quantitative analysis, statistics and advanced informatics (e.g., CytoSpeak package) were applied in this project to investigate the effector function (cytokine) landscape of single CAR-T cells. **d** A representative measurement of IFN-γ in supernatants from CAR-T cells stimulated by either IgG beads or anti-CAR beads by ELISA

To capture the full spectrum of complex T cell functions within the heterogeneous CAR-T population, we analyzed CAR-T cytokine production at the single cell level using a 16-plex panel. The 16-plex cytokine assay panel includes the key immune functions of T cells (e.g. effector, stimulatory, regulatory and inflammatory) (Fig. 1b & Additional file 3) and has been validated at both a population level and a single-cell level (Additional files 4 and 5). CAR-T cells generated from 4 different healthy donors were analyzed for cytokine secretion at the single cell level. The enriched CAR+ T cells were stimulated with anti-CAR beads or control IgG beads, stained with anti-CD4-PE and anti-CD8-AF647 and loaded into SCBC microchips for single cell cytokine analysis (Fig. 1a). Each of SCBC microchip device is comprised of a polydimethyl siloxane (PDMS) microchamber layer and a glass slide (see schematic in Fig. 1c **(i)**). The supporting glass slide is surface patterned with a miniaturized 16-element antibody microarray (Fig. 1c). The design of the SCBC permits optical microscopy inspection of the individual microchambers (Fig. 1c **(ii)**), including number of cells in each well and the cellular phenotypes (e.g., CD4^+^, CD8^+^, etc.) based on surface marker staining. The fluorescence based cytokine data is merged with the microscopy imaging data to generate the final data set. The number of cells, the cell phenotype and the cytokine production are therefore specified for each microchamber. We further adopted and/or developed advanced informatics tools (in IsoPlexis’ IsoSpeak software package) for not only statistical analysis of SCBC data sets but also the dissection of functional subsets (Fig. 1c **(iii)**). To evaluate the specificity of anti-CAR bead stimulation, CAR-T cells were stimulated with anti-CAR or IgG control beads in wells of 96-well plate for 24 h and the supernatant was analyzed by ELISA. Anti-CAR stimulation showed approximately 1000-fold increase of IFN-γ compared to control IgG beads stimulation (Fig. 1d), indicating the good specificity of in vitro anti-CAR bead stimulation. In addition, the increased cytokine secretion levels in CD19 CAR-T cells upon anti-CAR bead stimulation were observed across 4 donors compared to IgG control bead stimulation at a single-cell level, further demonstrating the stimulation specificity (Additional files 3 & 6).

### Increased polyfunctional heterogeneity of activated CD19 CAR-T cells dominated by an effector cytokine profile upon anti-CAR bead stimulation

**Fig. 2:**
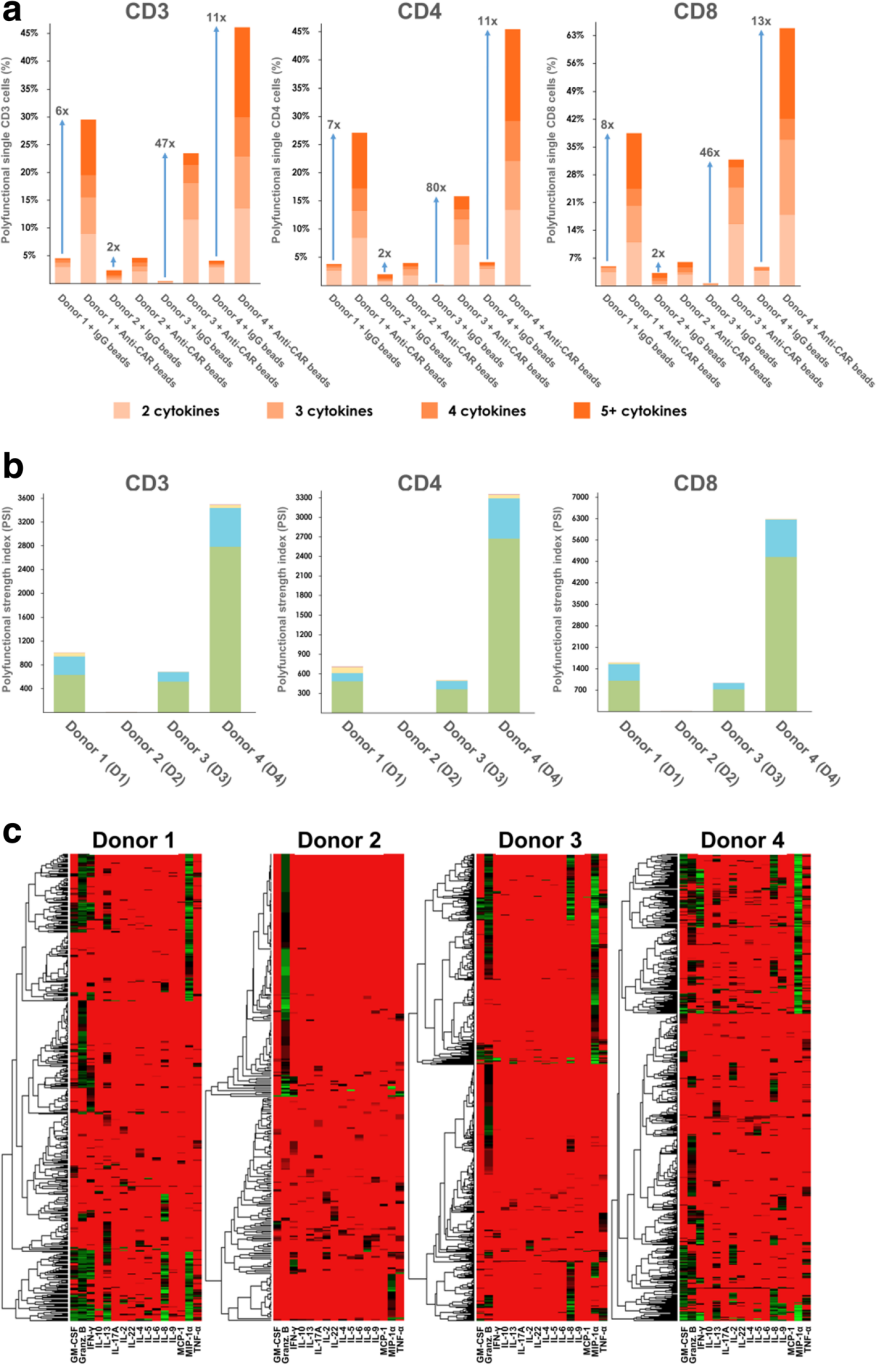
CAR-T cells show high polyfunctionality in anti-tumor effector and stimulatory functions. **a** Polyfunctional breakdown of CD3+, CD4+ and CD8+ T cells at the single-cell level across 4 donors. The T cells of donors 1, 3 and 4 show a 6 to 47-fold increase in overall polyfunctionality when stimulated with anti-CAR beads, compared to IgG stimulation. By comparison, donor 2 only shows a 2-fold increase. **b** Polyfunctional strength index (PSI) computed for CD3+, CD4+, and CD8+ T cells at the single-cell level across 4 donors. The profiles of donors 1, 3 and 4 is dominated by effector and stimulatory cytokine subsets. **c** Heat map and dendrogram visualization applied to the CD3 T cell secretion data. There is one heat map per donor, and each column corresponds to a single cytokine, while rows correspond to individual cells. Non-secreting cells are excluded from the heat maps. The colors indicate log transformed secretion intensities (red = low, green = high). At a high level, this visualization illustrates some differences across donors and which cytokines are commonly secreted in tandem. However, the clustering is done individually per donor, and it is difficult to map clusters to functional subsets

Given that the frequency of polyfunctional effector T cells correlates with potency of anti-tumor or anti-virus T cell immunity [24–26], we assessed the polyfunctionality of our CAR-T cell products. First, we computed the percentage of polyfunctional cells regardless of the combination of cytokines co-produced. As shown in Fig. 2a, under 5% of cells with IgG bead stimulation exhibited polyfunctionality. By contrast, the anti-CAR bead-stimulated CAR-T cells of donors 1–4 respectively showed a 6-, 2-, 47-, and 11-fold increase in polyfunctional cell counts for 4 donors, highlighting both the polyfunctionality and variability of CAR-T products across donors. In all 4 donors, we also noted that anti-CAR bead-stimulated CD8+ T cells were more polyfunctional than CD4+ T cells. Moreover, we used the polyfunctional strength index (PSI) described previously to quantify the collective impact of polyfunctional T cells [27]. The PSI of a sample is defined as the percentage of polyfunctional cells multiplied by the average signal intensity of the cytokines secreted by these cells. We further broke down PSI by cytokine function (Fig. 2b) – effector, stimulatory, regulatory, and inflammatory – to highlight the contribution of each group to the overall polyfunctionality of the sample. The PSI breakdown in Fig. 2b further revealed inter-donor heterogeneity of polyfunctional CAR-T cells. While effector and stimulatory cytokines contribute the most to polyfunctionality across all 4 donors, a small portion of regulatory and inflammatory cytokines were observed in donor 1 and donor 4. Furthermore, the observed regulatory and inflammatory response was mainly from the CD4+ T cells, consistent with the notion that both regulatory and helper T cells are subsets of CD4+ T cells, further proving the specificity of the SCBC assay. The donor-to-donor variability in polyfunctionality could be caused by a population-level shift of cytokine profiles, or the alteration of specific polyfunctional subpopulations. To better understand this variation, we first sought to use a conventional heatmap to visualize cytokine production from all single cells across four donors. The heat map data visualization in Fig. 2c gives a high-level indication that the analyzed single CAR-T cells exhibit significant differences in the combinations and intensities (red = low, green = high) of secreted proteins. However, it remains difficult to clearly visualize many functional subsets from heterogeneous CAR-T cells across donors.

### Limitations of conventional PCA and other standard visualizations in dissecting high-dimensional, single-cell, proteomic data of CAR-T cells

**Fig. 3:**
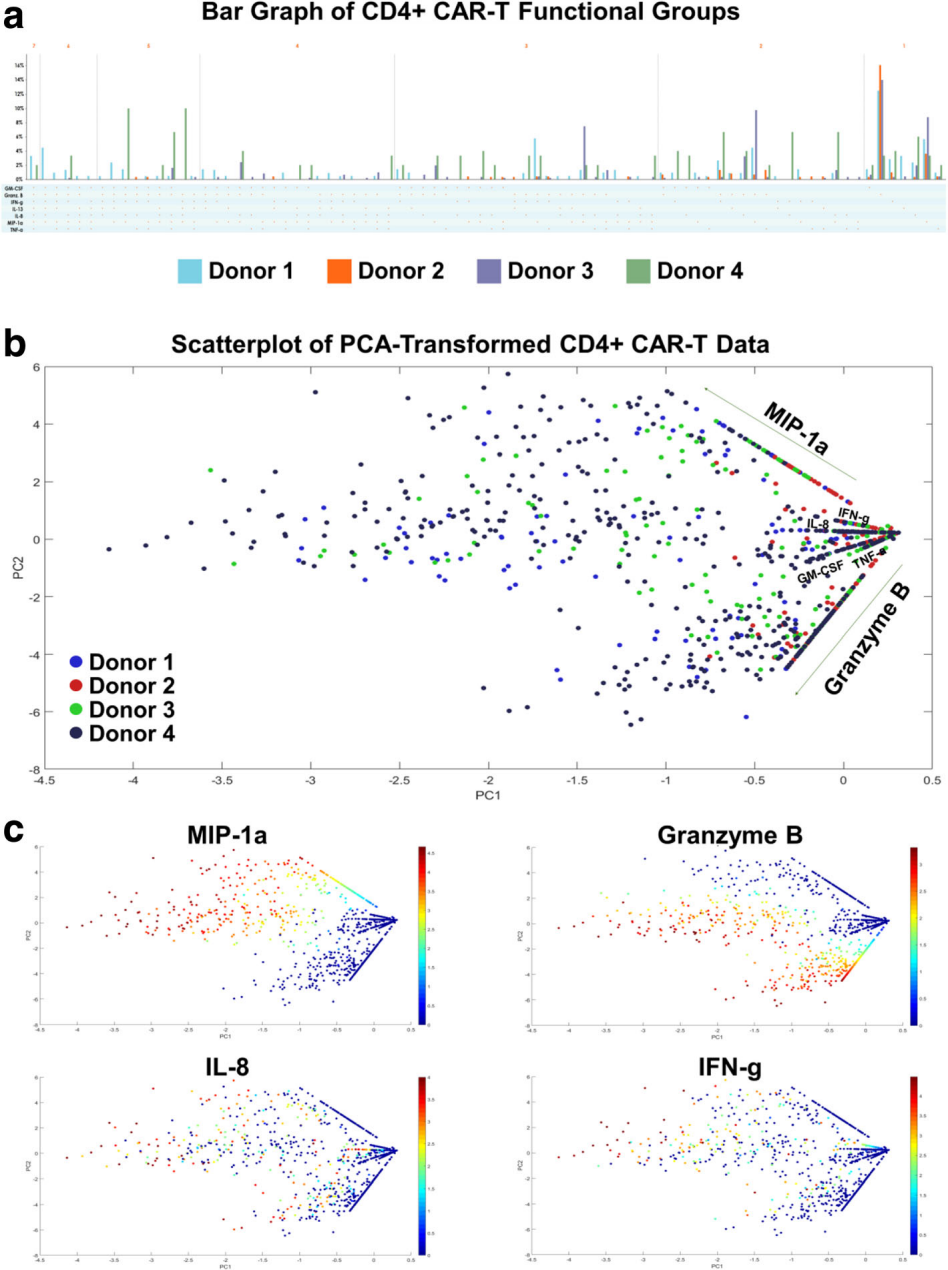
Higher dimensional data is difficult to visualize concisely. **a** With highly-multiplexed single-cell data, displaying the breakdown of different functional groups being secreted by a sample can increase by up to a factor of 2× with the addition of x cytokines. When this analysis is performed across a set of donors or stimulation conditions, effectively highlighting the key secretion differences is challenging. In this standard bar graph visualization of functional groups secreted by CD4+ CAR-T cells of four donors, it is cumbersome to see which are the major functional groups being secreted by each donor, and what are the biggest fold differences across donors. **b**-**c** Reducing the dimensionality of the dataset is a different approach to more effective and understandable visualizations. PCA (principal component analysis) uses an orthogonal transformation to convert the original dataset into a set of linearly uncorrelated principal components, where the number of components is smaller than the number of original variables. The transformation is defined in such a way that the first principal component has the largest possible variance (accounting for as much variability as possible within the dataset), followed by the second component, and so on. While reducing the dimensionality to two principal components may still result in some loss of information, the benefit is that the transformed data points can then be visualized on a two-dimensional scatterplot. In this figure, PCA is applied to the 4-donor CD4+ CAR-T secretion dataset. Each cell’s secretions (signal intensity of each cytokine) are log transformed prior to dimensionality reduction. **b** is color-coded by donor, while **c** is color-coded by some of the individual cytokines. The combination of these graphs reveals some information, such as the low overall polyfunctionality of donor 2, and the high Granzyme B + MIP-1a + polyfunctionality of Donor 4. However, more detailed information about upregulated and/or distinct polyfunctional subsets is less clear

We further explore other standard bioinformatics tools in visualizing this high-dimensional data set. In this standard bar graph visualization of the functional groups secreted by the four donors’ CD4+ CAR-T cells (Fig. 3a), the dimensionality of the data makes it cumbersome to see which are the major functional groups being secreted by each donor, as well as the largest fold differences across donors. An alternative approach to visualizing high-dimensional datasets is to first reduce the dimensionality of the data, while retaining as much of the original information as possible. PCA (principal component analysis) is a common dimensionality reduction technique which uses an orthogonal transformation to convert the original dataset of possibly correlated variables into a set of linearly uncorrelated principal components, where the number of components is smaller than the number of original variables. The transformation is defined in such a way that the first principal component has the largest possible variance (accounting for as much variability as possible within the dataset), followed by the second component, and so on. While reducing the dimensionality to two principal components may still result in some loss of information, the benefit is that the transformed data points can then be visualized on a two-dimensional scatterplot. Additionally, key differences within the transformed data should be magnified through this transformation. Figs. 3b-c displays the results of applying PCA to the 4-donor CD4+ CAR-T secretion dataset. Each cell’s secretions (signal intensity of each cytokine) are log transformed prior to dimensionality reduction. Fig. 3b shows a scatterplot of the transformed data color-coded by donor, while Fig. 3c shows the same data color-coded by some of the individual cytokines. The combination of these graphs does reveal additional information about donor response differences and expressed polyfunctional subsets, such as the lower overall polyfunctionality of donor 2, or the higher Granzyme B + MIP-1α + polyfunctionality of donor 1. A similar pattern of cytokine secretions was seen in the CD8+ CAR-T cells of all 4 donors (Additional file 7). However, two key issues still exist with this visualization: (1) it is difficult to infer which high-dimensional cytokine subsets are driving the polyfunctionality of each sample, and (2) it is unclear what are the more granular polyfunctional differences across the analyzed donor samples. Other methods such as viSNE (Additional file 8) that map high-dimensional cytometry data onto two dimensions, yet conserve the high-dimensional structure of the data, improve the ability to distinguish cell subpopulations [28, 29]. However, the polyfunctional breakdown of the samples remains unclear when this visualization is used. In the context of polyfunctional T cell analysis, the routine practice has been to manually enumerate the major polyfunctional subsets by quantifying the cell count in each of the possible cytokine combinations, which serves well the ability to distinguish polyfunctional T cell subsets but loses the relationship between subsets or the hierarchical structure of the population. We hereby propose two alternative visualizations, polyfunctional heat map and polyfunctional activated topology principal component analysis (PAT PCA) which in tandem work to solve these problems.

### Polyfunctional heat map to highlight distinct polyfunctional T cell subsets and the heterogeneity within a cell population

**Fig. 4:**
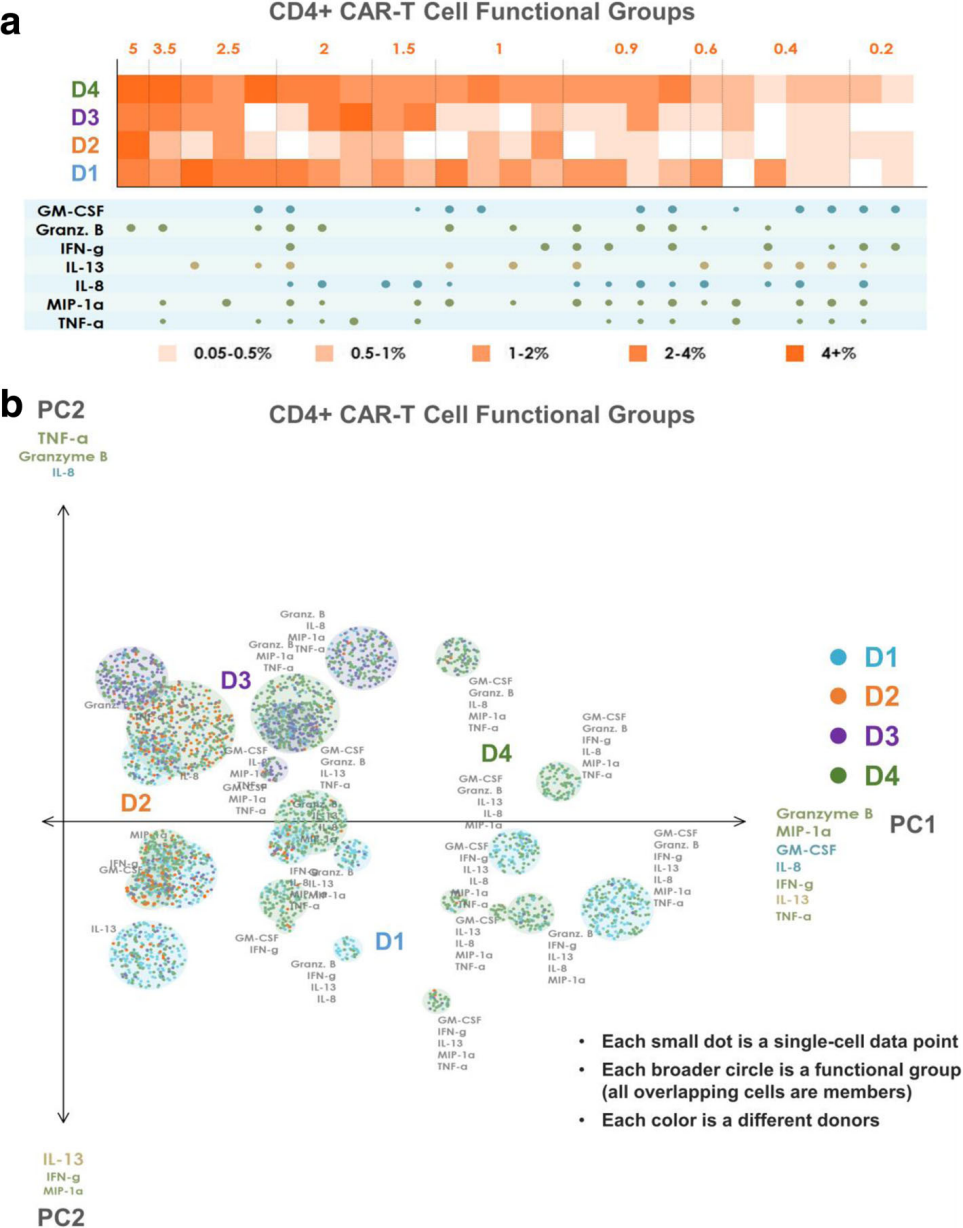
Polyfunctional heat map and PAT PCA reveal distinct CD4+ CAR-T cell profiles across donors. **a** Polyfunctional heat map displaying major functional subsets secreted across the 4 donors’ CD4+ CAR-T samples. Hierarchical clustering is applied to attain a condensed set of functional groups that still faithfully represent the overall profile of the donors. The color-coding indicates how commonly each donor secrets the corresponding functional group/cluster. Donor 1, closely followed by donor 4, has the highest frequencies of most expressed functional groups. Donor 3 is less polyfunctional, while donor 2 has virtually no secreted polyfunctional groups. The group GM-CSF, Granzyme B, IL-13 and TNF-α is expressed exclusively by the CD4+ CARs of donors 1 and 4, but not by the CARs of donor 2 or donor 3. Similarly, the 7-plex group containing GM-CSF, Granzyme B, IFN-γ, IL-8, IL-13, MIP-1α, and TNF-α is unique to these two donors. Functional groups not containing GM-CSF or IL-13 are expressed at similar frequencies by donor 3 as they are by donors 1 and 4. **b** PAT PCA visualization of the same dataset. Data points are color-coded based on donor. Those representing the same functional group are randomly offset, but remain within a radius proportional to the secretion frequency of the corresponding group (i.e., large groups = large circles, small groups = small circles). The principal components are labeled according to their correlation with specific cytokines. The lack of donor 2 (orange) subsets indicates the lower polyfunctionality of this sample, while the presence of numerous donor 1 (blue) and 4 (green) groups in the right area of the graph indicates the highly-polyfunctional makeup of these two samples. Donor 3 has generally less polyfunctional subsets, typically including combinations of Granzyme B, MIP-1α, IL-8, and TNF-α but lacking IFN-γ, IL-13, and GM-CSF. Donor 4 largely spans the polyfunctional profiles of both donors 1 and 3

**Fig. 5:**
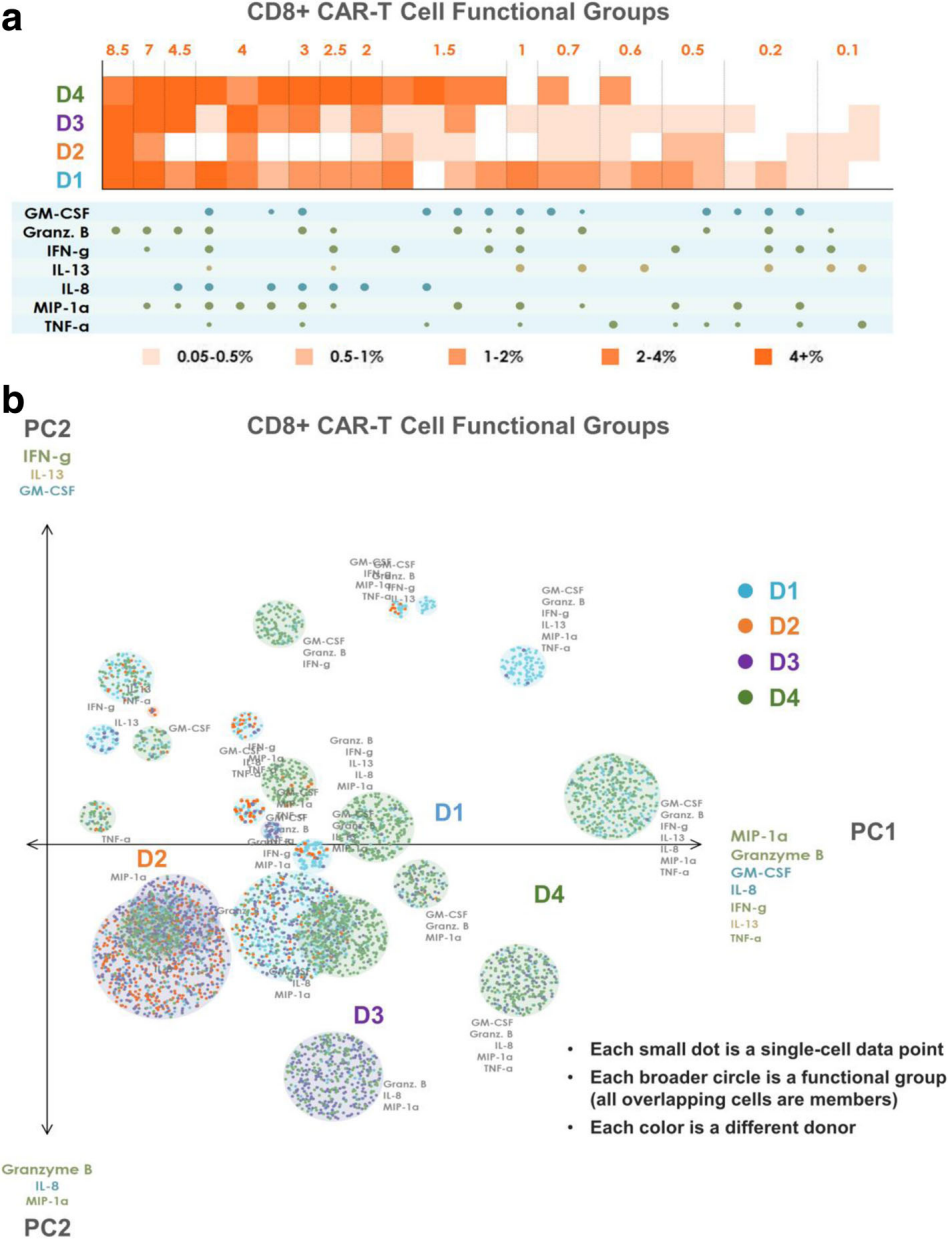
Polyfunctional heat map and PAT PCA reveal distinct CD8+ CAR-T cell profiles across donors. **a** Polyfunctional heat map displaying major functional subsets secreted across the 4 donors’ CD8+ CAR-T samples. Hierarchical clustering is applied to attain a condensed set of functional groups that still faithfully represent the overall profile of the donors. The color-coding indicates how commonly each donor secrets the corresponding functional group/cluster. Donors 1 and 4 are significantly more polyfunctional than donors 2 and 3, and also uniquely secrete the 7-plex group containing GM-CSF, Granzyme B, IFN-γ, IL-8, IL-13, MIP-1α, and TNF-α. Donor 1 has a higher number of unique polyfunctional groups than donor 4, particularly groups not containing IL-8. The only polyfunctional groups secreted by all four donors contain Granzyme B, MIP-1 α with smaller amounts of IFN- γ. **b** PAT PCA visualization of the same dataset. Data points are color-coded based on donor. Those representing the same functional group are randomly offset, but remain within a radius proportional to the secretion frequency of the corresponding group (i.e., large groups = large circles, small groups = small circles). The principal components are labeled according to their correlation with specific cytokines. Like the CD4+ CAR-T samples, donor 2 (orange) has low polyfunctionality, donors 1 (blue) and 4 (green) are highly polyfunctional. Donor 3 has generally less polyfunctional subsets often comprised of combinations of Granzyme B, MIP-1α, IL-8, and TNF-α but lacks IFN-γ, and IL-13. Donor 4 largely spans the polyfunctional profiles of both donors 1 and 3, which can also be seen in the heat map

To distinguish all polyfunctional subsets within a sample and dissect the population architecture, we developed a new polyfunctional heat map visualization. This visualization, shown in Fig. 4a for CD4+ CAR-T cells and Fig. 5a for CD8+ CAR-T cells, displays the major functional subsets secreted across the 4 donor samples. The heat maps are color-coded from light to dark, depending on the frequency of the polyfunctional cell subsets. The four rows of squares correspond to the four donors; each column corresponds to a polyfunctional group of cytokines that was expressed in at least one of the four samples. To condense the large number of functional groups arising from the high dimensionality of the data set, we use agglomerative hierarchical (complete linkage) clustering to attain a condensed set of functional groups that still faithfully represent the overall secretion profile of the donors. Using a Euclidean distance to measure similarity between functional groups, each unique group is input into the clustering algorithm as a 16-dimensional vector of 1 s and 0 s, corresponding to the presence or absence, respectively, of each cytokine in the group. We define the minimum permitted similarity value to perform a clustering operation, to ensure that clusters do not contain functional groups that are too distinct from each other. The resulting clusters and their frequencies are displayed in the functional heat map, with the size of the cytokine dots below each column representing the frequency of the corresponding cytokine in the cluster. As seen in Fig. 4a, donor 1, closely followed by donor 4, has the highest frequencies of most expressed functional groups. Donor 3 is less polyfunctional, while donor 2 has the least polyfunctional groups. The group GM-CSF, Granzyme B, IL-13 and TNF-α is expressed exclusively by the CD4+ CARs of donors 1 and 4, but not by the CARs of donor 2 or donor 3. Similarly, the 7-plex group containing GM-CSF, Granzyme B, IFN-γ, IL-8, IL-13, MIP-1α, and TNF-α is unique to these two donors, revealing functional differences of these CAR cells relative to those of donors 2 and 3 at a high degree of granularity. Functional groups not containing GM-CSF or IL-13 are expressed at similar frequencies by donor 3 as they are by donors 1 and 4. As seen in Fig. 5a, the CD8+ CAR cells similarly show donors 1 and 4 to be significantly more polyfunctional than donors 2 and 3, and also uniquely secrete the 7-plex group containing GM-CSF, Granzyme B, IFN-γ, IL-8, IL-13, MIP-1α, and TNF-α. Donor 1 has a higher number of unique polyfunctional groups than donor 4, particularly groups not containing IL-8. The only polyfunctional groups secreted by all four donors contain Granzyme B, MIP-1 α with smaller amounts of IFN- γ.

### PAT PCA to further visualize distinct polyfunctional T cell subsets and a complex landscape of CD19-specific immune response

In addition to the heat map visualization, we propose a modified PCA visualization named PAT PCA illustrated in Additional file 2. We use this visualization to help reveal a complex landscape of polyfunctional subsets of CAR-T cells in response to antigen-specific stimulation across donors. Figs. 4b and 5b show PAT PCA visualizations of the same single-cell-resolution CD4+/CD8+ CAR results as the heat maps in Figs. 4a and 5a, respectively. Similarly for the hierarchical clustering input, we apply PCA on a binarized dataset (0 = no secretion, 1 = secretion), to focus on visualizing combinatorial differences, rather than intensity differences. In the resulting scatterplot visualization, each color-coded dot represented a single-cell from one of the four donors, respectively, in which the larger color-coded circles represented a unique polyfunctional subset. The colors of the subsets identified the sample where this subset was most frequent (i.e., largest as a percentage of the sample). The functional groups (columns) in the heat map are represented by a circle in the corresponding PAT PCA graph. The size of the circles corresponds to the frequency of the group; the number of cells (dots) within each group indicates how frequently the sample of the corresponding color secreted the group. The makeup of the two principal components PC1 and PC2 is indicated by the listed cytokines, with the composition of PC1 indicating the main drivers of polyfunctionality. In both graphs, PC1 is a combination of the seven dominant secretors of the anti-CAR stimulated cells: effector cytokines Granzyme B, MIP-1α, IFN-γ, and TNF-α; stimulatory cytokines GM-CSF and IL-8; and the regulatory cytokine IL-13. As a result, the highly-polyfunctional groups are farther along (on the right-side of) this axis. PC2 differed between CD4 and CD8 cells. In the CD4 case, groups towards the top tend to have more Granzyme B, IL-8 and TNF-α, while groups near the bottom more often contain IL-13, IFN-γ and MIP-1α. In the CD8 case, groups near the top more commonly secreted IFN-γ, IL-13 and GM-CSF, while groups near the bottom more commonly secreted Granzyme B, IL-8 and MIP-1α. Plotting the polyfunctional subsets in such a manner allows overall similarities and differences in the donor profiles to emerge. The lack of donor 2 (orange) subsets indicated the lower polyfunctionality of this sample, while the presence of numerous donor 1 (blue) and donor 4 (green) groups in the right area of the graph indicated the highly-polyfunctional makeup of these two samples. Donor 3 has fewer polyfunctional subsets mostly comprised of combinations of Granzyme B, MIP-1α, IL-8, and TNF-α but lacking IFN-γ, IL-13, and in the case of CD4+ cells, GM-CSF. Donor 4 largely spans the polyfunctional profiles of both donors 1 and 3, which can also be seen in the heat maps. Capturing the full polyfunctional landscape of each sample is critical to effectively analyzing this data, as well as identifying the major subsets contributing to differences between samples/donors. The presented polyfunctional heat map and PAT PCA graphs are effective at achieving these objectives, both individually and together, and provide the first landscape of effector function phenotypes of CD19 CAR-T cells in response to antigen-specific challenge.

## Discussion

Defining clinical relevant functional attributes of CAR-T products has been a primary objective of pre-infusion analytics strategy of CAR-T cell therapy, yet key attributes that are associated with efficacy or safety are still unknown [30]. This is mainly due to two reasons. Firstly, CAR-T final product is a complex live cell population with thousands of unique phenotypes and myriad functions that peripheral blood derived T cells possess [31]. Individual CAR-T cell may respond in a markedly different manner to antigen specific stimulation as defined by a host of immune effector cytokines secreted. Secondly, the most commonly utilized assays to assess CAR-T functions in the context of CD19 CAR-T therapies are cytotoxicity assay and interferon gamma release assay [30]. In both cases, CAR-T cells are co-cultured at a population level with target cells, a cell line that expresses CAR specific tumor antigen. Measuring one averaged signal, both assays fail to take into account the full diversity of T cell functions and are not adequate to provide complete data for clinical correlations [30]. As such, a highly multiplexed, single cell approach is needed for comprehensive pre-infusion assessment of CAR-T product protein secretion to enable biomarker development. We propose the SCBC platform, which measures a broad range of up to 45 secreted proteins at the single cell level, in combination with the newly developed polyfunctionality analysis software, is well suited to address this pressing need. In this study, we measured 16 immune functions including anti-tumor effector, stimulatory, inflammatory, and regulatory cytokines at the single-cell level on human CD19 CAR-T cells (Fig. 1). An elevated cytokine production with dominant anti-tumor effector profile from CD19 CAR-T cells in response to antigen-specific stimulation was observed across all 4 donors (Fig. 2 and Additional file 3). Polyfunctional subsets were further analyzed and visualized using newly developed bioinformatics tools, polyfunctional heat map and PAT PCA (Figs. 4 and 5). All together, we demonstrated that the SCBC platform represents a new platform that enables a comprehensive profiling of CAR-T cell cytokine/chemokine production and supports correlative analysis for biomarker discovery in pre-infusion CAR-T products.

Initial evidence suggests that polyfunctional CAR-T cells may serve as a useful biomarker for efficacy. Dynamic polyfunctionality analysis of adoptively transferred MART-1-specific TCR-engineered T cells showed that TNF-α+IFN-γ+ polyfunctional T cell delayed tumor relapse [27]. Furthermore, a study comparing in vitro functions of CD19 CAR-T cells generated from different CAR structures showed that polyfunctionality rather than cytotoxicity of the CAR-T cells was a better predictor of therapy efficacy in vivo [32]. The observation that persisting CAR-T cells in CLL complete responders remain polyfunctional 4 years after infusion suggests an association between polyfunctional CAR-T cells with persisting response [9]. The significance of T cell polyfunctionality in product characterization as well as clinical biomarkers led us to bring this metric to the forefront of our data analysis and visualizations. We first quantitated the level of polyfunctionality using PSI analysis which results in a polyfunctionality score for each donor (Fig. 2a and b). Overall, an increase in polyfunctionality of CAR-T cells in response to anti-CAR bead stimulation was observed in all four donors, with significant donor-to-donor heterogeneity (Fig. 2). CAR-T cells from donors 1 and 4 have the highest polyfunctionality, whereas CAR-T cells from donor 2 have the least. The polyfunctionality was predominated by effector cytokine profiles, which is consistent with the high potency observed with CD19 CAR in previous clinical trials.

We then sought to breakdown the unique combinations of cytokine subsets secreted by a sample. The further analysis requires bioinformatics tools as the number of possible subsets can increase by up to a factor of 2^n^ with the addition of n cytokines. On top of this, when analyzing differences across a set of donors or stimulation conditions, effectively highlighting the key polyfunctional differences is challenging. Previously developed single cell analysis bioinformatics tools, however, do not fit the purpose of polyfunctional analysis, especially for the comparison between multiple donors [29, 33]. For example, conventional heatmap can indicate heterogeneity among single cells within one donor at a high level, but it cannot map functional subsets of different samples in the same space, therefore making multi-donor comparison very difficult (Fig. 2c). While conventional PCA and viSNE can map multiple donors in the same space, neither can identify polyfunctional subsets that drive the differentiations between donors at a high level of granularity (Fig. 3 and Additional file 8). To address this need, we developed two informatics visualization tools, namely a polyfunctional heat map and a PAT PCA scatterplot visualization, which are able to capture the full landscape of complex polyfunctional subsets within each donor, and identify major subsets that are different between donors and the driving factors for these differences. For example, polyfunctional heatmap showed while all four donors have polyfunctional subsets secreting Granzyme B, MIP-1α and IFN-γ, only donor 1 and donor 4 have a highly polyfunctional subsets secreting GM-CSF, Granzyme B, IFN-γ, IL-8, IL-13, MIP-1α and TNF-α. PAT PCA visualization further reveals that the polyfunctional subsets of donor 3 are driven mainly by IL-8, Granzyme B, TNF-α (CD4) and MIP-1α (CD8) whereas those of donor 1 typically include IFN-γ and IL-13. Overall, we saw a convincing increase in the clarity of polyfunctional heatmaps and PAT PCA over other state of the art visualizations, demonstrating the overarching importance of developing visualizations that highlight polyfunctionality in sample characterization and differentiation. As with all novel, high dimensional data, one key challenge is how to effectively use the data. Future studies will explore other extensions and optimizations to the presented polyfunctionality visualizations with larger datasets.

The presented single cell analysis platform is spun out from previous academic prototype with rigid validations and significant upgrades [18]. The current data acquisition and analysis platform features: (1) measurements of true protein secretion from single cells residing in a nanowell, representing a more physiological environment than any other available single cell technology. (2) an automated acquisition process with data integrity and minimal user interference, and (3) an integrated bioinformatics suite for understanding and highlighting key differences in donor response (Fig. 1). The readout of the assay is fluoresce intensity of accumulated cytokines over the 16 h incubation time. Protein concentrations may be inferred using recombinant protein standard curves that generated on a separate or same chip [34]. Temporal protein secretions of single cells for time-point study can be measured by analyzing a serial of chips. So far, the platform has been applied on a broad range of studies, including cytokine paracrine signaling analysis, malignant cell characterization and vaccine evaluation in infectious disease [34–36]. Here, we extended the application to CAR-T analysis, demonstrating its capability to comprehensively characterize CAR-T cell pre-infusion products. Future studies on patient sample analysis, in combination with correlative studies with clinical responses will likely provide meaningful data for biomarker identification.

How a patient responds to CAR-T therapy is affected by multiple factors, the function of CAR-T cells, the phenotype of tumor cells, the engagement of other immune cells, etc. Although the current study provides in-depth functional analysis of CAR-T cells, other contributing factors may confound the correlation between CAR-T functions with clinical responses. To alleviate the confounding effect of tumor cell variations across patients, a future experiment is to include single CAR-T cells with autologous tumor cells in the same microchamber to evaluate patient-specific CAR-T response. In fact, the presented SCBC system has been previously applied to the study of cell-cell interactions by including multiple cells in one microchamber [37, 38]. The fact that only a small number of cells (~ 1 × 10^4^ cells) are required for setting up one SCBC chip makes the presented technology especially suitable for CAR-T autologous tumor response studies. In addition, nanowell-based single cell technologies have been previously used to study cytotoxicity of single NK cell or CAR-T cell in response to target cells [39, 40]. By incorporating cell viability staining and microscopy imaging, a single cell autologous cytotoxicity assay can be built into the SCBC system, enabling simultaneously measurement of cytotoxicity and cytokine/chemokine production from one single cell.

## Conclusions

We have demonstrated how single-cell multiplexed proteomics is able to comprehensively characterize CD19 CAR-T cell product cytokine/chemokine profiles. Our single-cell analysis has, for the first time, revealed the multifunctional heterogeneity of CD19 CAR-T cell products by antigen-specific stimulation. Through the SCBC, we measured CAR-T cell secretion profiles across a panel of 16 key immune functions, and noted clear distinctions in the range of single-cell responses across each of four donors. We additionally developed new polyfunctional heat map and PAT PCA visualizations to deconvolute single-cell-level differences across donor responses, and to provide an in-depth understanding of the polyfunctional subsets driving each donor’s responses. Although the result presented here are based on CAR-T cells from healthy donor materials, it illustrates the necessity and provide a possible blueprint for guiding CAR-T product pre-infusion assessment. The ability to precisely dissect single-cell, polyfunctional heterogeneity supports a road map for correlative discoveries on the role of polyfunctional CAR-T cells in clinical responses and has the potential to greatly impact CAR-T therapy optimization.

## Additional files

Additional file 1: Table S1. 16-Plex SCBC Antibody Panel. (PDF 2103 kb)

Additional file 2: Figure S3. Overview of data transformations for PAT PCA. A: An illustrative example showing how raw single-cell data (signal intensities) from a single-cell multiplex cytokine assay are transformed. The polyfunctional group of each cell is found along with unique groups (encoded by a vector of 0 s and 1 s) and their frequencies. An adjusted frequency n, weighted by polyfunctionality, is computed per group. Each vector is included n times and this resulting dataset is transformed using PCA. (PDF 2103 kb)

Additional file 3: Figure S1. CAR-specific stimulation induces multiple cytokine production at the single-cell level. Cytokine secretions of total CD3 T cells, CD4 T cells and CD8 T cells, all stimulated by anti-CAR beads, are shown across 4 donors and compared to the control secretion profile. The analyzed 16-plex panel includes 4 color-coded groups of cytokines: effector (green), stimulatory (blue), regulatory (yellow) and inflammatory (red). Low secretion percentages, as well as secretions with an average signal noise ratio (SNR) < 2 are labeled not significant (gray). (PDF 2103 kb)

Additional file 4: Figure S6. Validation of the antibodies in the 16-plex single-cell panel. (A) standard RFU/protein curve for the 16-plex panel. Antibody pairs from multiple manufacturers were tested for sensitivity with recombinant protein by titrating recombinant protein cocktails (5, 15.8, 50, 158, 500, 1580 and 5000 pg/mL) to produce a standard RFU/protein curve. (B) Antibody pairs were tested for specificity by spiking 1000 pg/mL protein standards for each antibody on the panel. Antibody pairs were then evaluated for cross reactivity within the panel. Antibodies were considered specific when the antibody pair had an SNR >10. (PDF 2103 kb)

Additional file 5: Figure S7. Validation of the 16-plex cytokine panel on the SCBC platform. (A) A representative signal distribution of Granzyme B, IFN-γ and TNF-α from single CD8 T cells at the SCBC platform. (B) A representative ICS data of IFN-γ and TNF-α secreting CD8 T cells. (C) A pooled comparison data of IFN-γ and TNF-α secreting CD8 T cells between SCBC and ICS. (D) The correlation of 16 protein secretion levels between single-cell averages from two independent experiments (x, y axes: % of cytokine-secreting single CD8 T cells). (E) A representative scatter plots of Granzyme B and IL-8 from individual experiments. (PDF 3044 kb)

Additional file 6: Figure S2. The level of cytokine secretion from single cells and populations upon anti-CAR bead stimulation of CD19 CAR-T cells. At both the single-cell level and bulk-level, an overall increase in the intensity of effector and stimulatory cytokine secretions was observed with anti-CAR bead stimulation (orange) compared to control IgG bead stimulation (blue). While bulk-level measurements only show an average intensity per cytokine of the entire cell sample, single-cell level measurements present a full distribution of cell-by-cell secretion intensities. Levels of upregulation are consistent between the bulk-level measurement and single-cell level measurement across donors, with donor 2 having very small increases compared to the other three donors at both levels. (PDF 2103 kb)

Additional file 7: Figure S4. Higher dimensional data is difficult to visualize concisely. (A) In this standard bar graph visualization of functional groups secreted by CD4+ CAR-T cells of four donors, it is cumbersome to see which are the major functional groups being secreted by each donor, and what are the biggest fold differences across donors. (B-C) Reducing the dimensionality of the dataset is a different approach to more effective and understandable visualizations. In this figure, PCA is applied to the 4-donor CAR-T secretion dataset. Each cell’s secretions (signal intensity of each cytokine) are log transformed prior to dimensionality reduction. (B) is color-coded by donor, while (C) is color-coded by some of the individual cytokines. The combination of these graphs reveals some information, such as the low overall polyfunctionality of donor 2, and the high Granzyme B+MIP-1a+ polyfunctionality of Donor 4. However, more detailed information about upregulated and/or distinct polyfunctional subsets is less clear. (PDF 2103 kb)

Additional file 8: Figure S5. viSNE visualization of CD4+ CAR-T data. viSNE is a visualization tool designed to map high-dimensional flow cytometry data onto two dimensions, while preserving the overall structure of the data. Similar to PCA, color can be used as a third dimension in the resulting visualization. In this figure, color is used to indicate (A) the donor sample of each single CD4+ CAR-T cell or (B) the intensity of individual cytokine secretions of each CD4+ CAR-T cell. Unlike PCA, which is a linear transformation, the benefit of visNE is its ability to preserve non-linear relationships across the data. One can infer that a subset of cells in each donor secrete only Granzyme B, that primarily donors 3 and 4 have cells secreting only TNF-a, and that donors 1 and 4 both have unique subsets of highly polyfunctional, Granzyme B + MIP-1a + IFN-g + secreting cells. However, additional donor differences and specific information about functional groups is fairly limited. A viSNE transformation of the CD8+ CAR-T data gives a similar graph. (PDF 2866 kb)

## Abbreviations

B-ALL: B-cell precursor acute lymphocytic leukemia
CAR: Chimeric antigen receptor
CLL: Chronic lymphocytic leukemia
CRS: Cytokine release syndrome
DLBCL: Diffuse large B-cell lymphoma
ICS-FC: Intracellular staining flow cytometry
PAT PCA: Polyfunctional activation topology principal component analysis
PBMC: Peripheral blood mononuclear cell
PDMS: Polydimethyl siloxane
PSI: Polyfunctional strength index
SCBC: Single-cell barcode chip
