# Supplementary material for: Single-cell multiplexed cytokine profiling of CD19 CAR-T cells reveals a diverse landscape of polyfunctional antigen-specific response
Source: J Immunother Cancer. 2017 Nov 21;5:85. doi: 10.1186/s40425-017-0293-7 (PMC5697351; doi:10.1186/s40425-017-0293-7)
Supplement: Supplementary file 1 — 16-Plex SCBC Antibody Panel. (PDF 2103 kb) [file 40425_2017_293_MOESM1_ESM.pdf]

Additional file 1 (Table S1)

	Cytokine	Capture	Detection
**1**	TNF-α	eBioScience 88-7346	eBioScience 88-7346
**2**	IFN-γ	eBioScience 14-7318	eBioScience 13-7319
**3**	IL-5	eBioScience 88-7056	eBioScience 88-7056
**4**	IL-8	eBioScience 14-7086	eBioScience 33-8088
**5**	IL-9	eBioScience 88-7958	eBioScience 88-7958
**6**	IL-10	eBioScience 88-7106	eBioScience 88-7106
**7**	IL-4	eBioScience 88-7046	eBioScience 88-7046
**8**	IL-6	eBioScience 88-7066	eBioScience 88-7066
**9**	IL-17A	eBioScience 88-7176	eBioScience 88-7176
**10**	IL-22	eBioScience 88-7522	eBioScience 88-7522
**11**	IL-2	eBioScience 88-7025	eBioScience 88-7025
**12**	GM-CSF	eBioScience 88-8337-88	eBioScience 88-8337-88
**13**	MIP-1α	eBioScience 88-7035	eBioScience 88-7035
**14**	MCP-1	eBioScience 88-7399	eBioScience 88-7399
**15**	Granzyme B	MabTech 3485-3-250	R&D SEL2906
**16**	IL-13	eBioScience 88-7439	eBioScience 88-7439
