# Supplementary material for: Single-cell multiplexed cytokine profiling of CD19 CAR-T cells reveals a diverse landscape of polyfunctional antigen-specific response
Source: J Immunother Cancer. 2017 Nov 21;5:85. doi: 10.1186/s40425-017-0293-7 (PMC5697351; doi:10.1186/s40425-017-0293-7)
Supplement: Supplementary file 2 — Overview of data transformations for PAT PCA. A: An illustrative example showing how raw single-cell data (signal intensities) from a single-cell multiplex cytokine assay are transformed. The polyfunctional group of each cell is found along with unique groups (encoded by a vector of 0 s and 1 s) and their frequencies. An adjusted frequency n, weighted by polyfunctionality, is computed per group. Each vector is included n times and this resulting dataset is transformed using PCA. (PDF 2103 kb) [file 40425_2017_293_MOESM2_ESM.pdf]

Additional file 2 (Figure S3)

Data Transformation: **Example:**

Raw single-cell data

	MIP-1a	IL-8	Gran. B
**Cell 1**	200	50	200
**Cell 2**	100	200	250
**Cell 3**	120	50	300

↓

Background-subtracted data

	MIP-1a	IL-8	Gran. B
**Cell 1**	160	0	50
**Cell 2**	60	150	100
**Cell 3**	80	0	150

↓

Cell polyfunctionality

	MIP-1a	IL-8	Gran. B	Polyfunc.	Group
**Cell 1**	1	0	1	2	Group 1
**Cell 2**	1	1	1	3	Group 2
**Cell 3**	1	0	1	2	Group 1

↓

Unique groups

	MIP-1a	IL-8	Gran. B	Polyfunc.	Freq.	Adj. Freq.
**Group 1**	**1**	**0**	**1**	2	2	2*2 = 4
**Group 2**	**1**	**1**	**1**	3	1	3*1 = 3

↓

PCA-transformed groups

	x = PC1	y = PC2
**Group 1**	0.5	0.2
**Group 2**	0.7	-0.4

Each 3-dimensional vector included at adjusted frequency; dataset (8 points) transformed using PCA

↓

Offset single-cell locations

	x = PC1	y = PC2
**Cell 1**	0.51	0.21
**Cell 2**	0.7	-0.4
**Cell 3**	0.49	0.19

Cell 1 and Cell 3 offset for visibility due to identical initial coordinates; both remain within bounds of Group 1 circle
