# Supplementary figures and images for: Single-cell multiplexed cytokine profiling of CD19 CAR-T cells reveals a diverse landscape of polyfunctional antigen-specific response
Source: J Immunother Cancer. 2017 Nov 21;5:85. doi: 10.1186/s40425-017-0293-7 (PMC5697351; doi:10.1186/s40425-017-0293-7)

Additional file 3 (Figure S1)

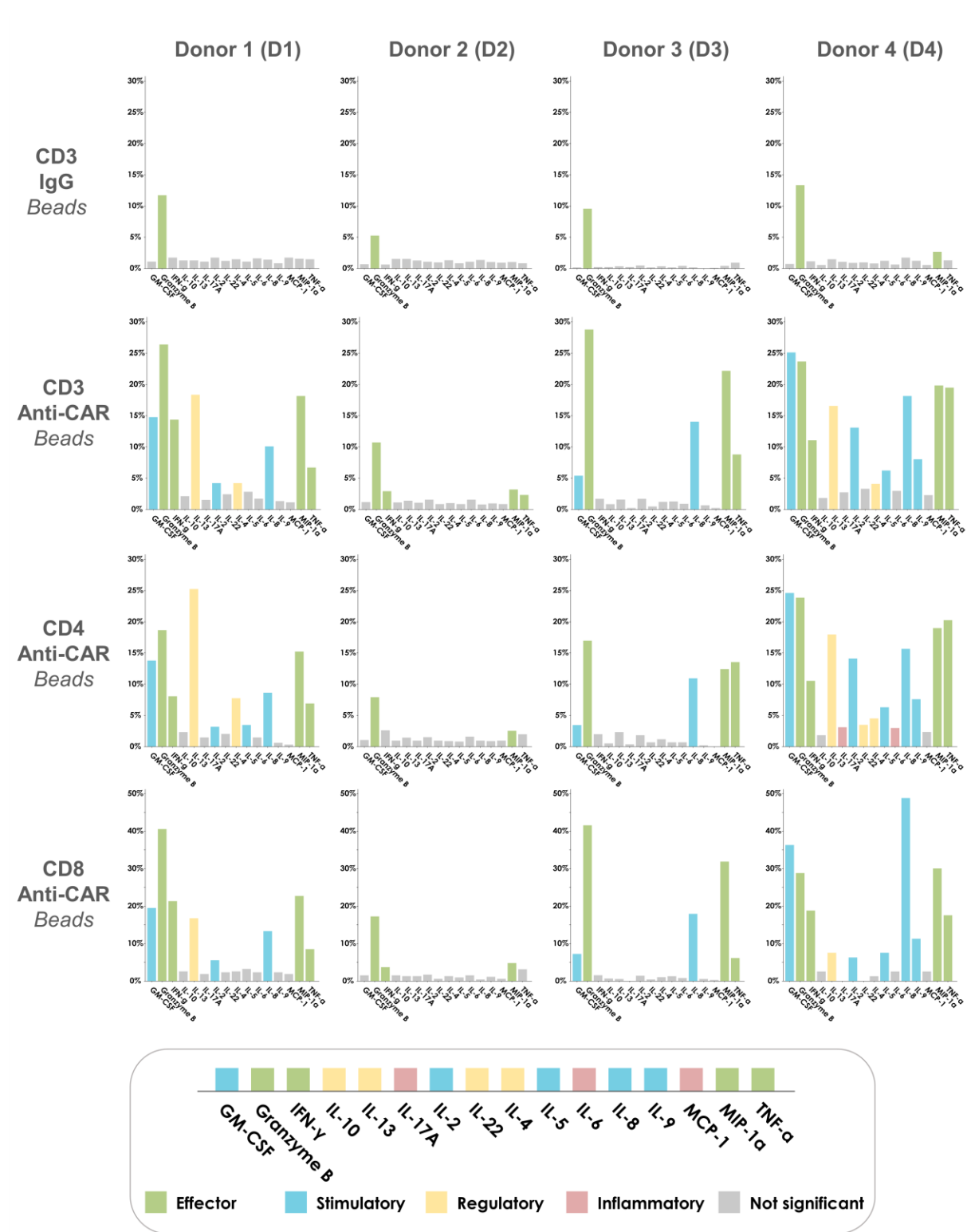

Supplement: Supplementary file 3 — CAR-specific stimulation induces multiple cytokine production at the single-cell level. Cytokine secretions of total CD3 T cells, CD4 T cells and CD8 T cells, all stimulated by anti-CAR beads, are shown across 4 donors and compared to the control secretion profile. The analyzed 16-plex panel includes 4 color-coded groups of cytokines: effector (green), stimulatory (blue), regulatory (yellow) and inflammatory (red). Low secretion percentages, as well as secretions with an average signal noise ratio (SNR) < 2 are labeled not significant (gray). (PDF 2103 kb) [file 40425_2017_293_MOESM3_ESM.pdf]

Additional file 4 (Figure S6)

A

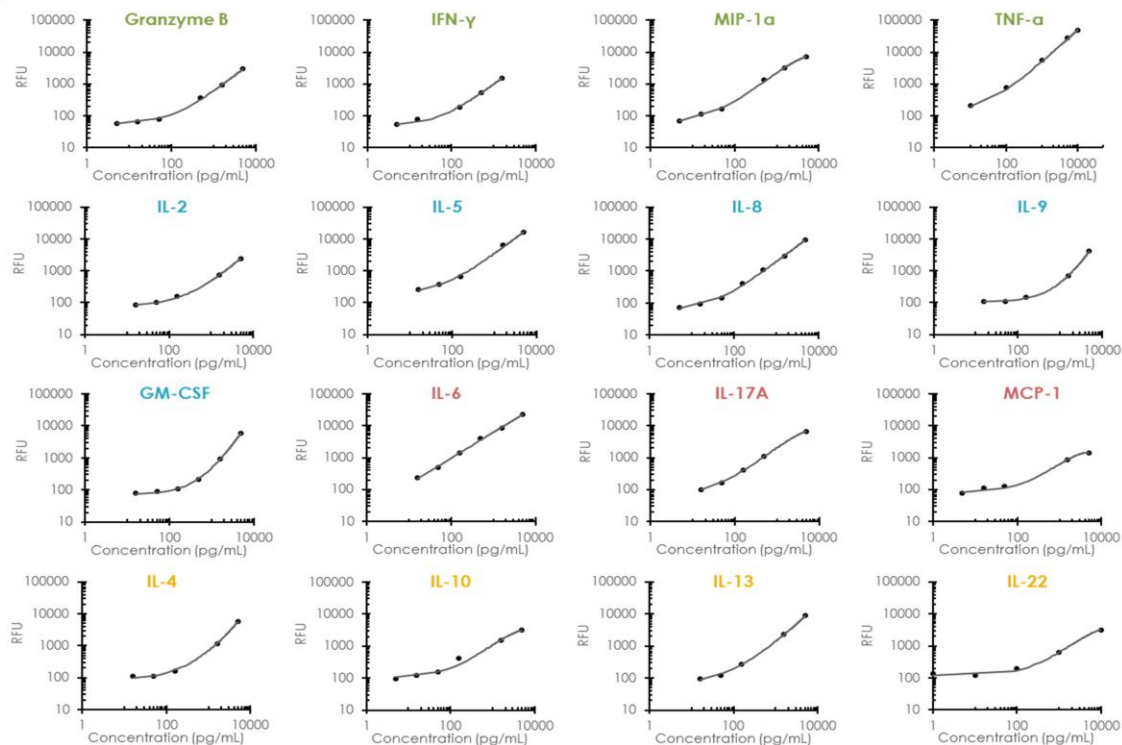

B

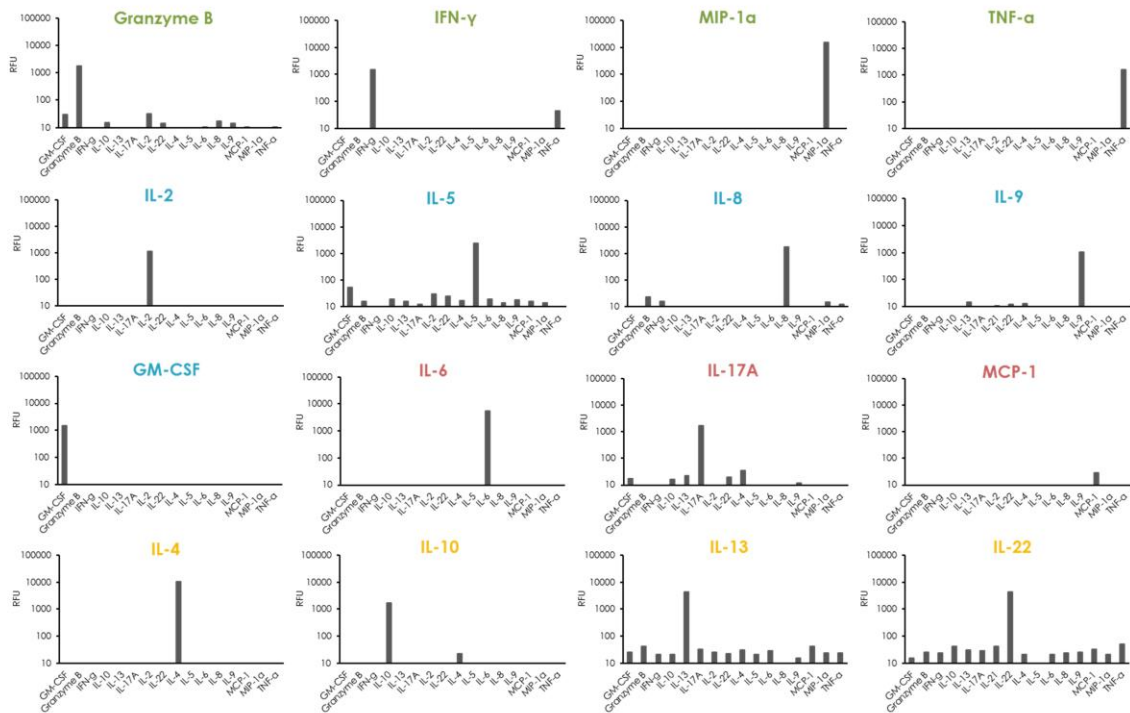

Supplement: Supplementary file 4 — Validation of the antibodies in the 16-plex single-cell panel. (A) standard RFU/protein curve for the 16-plex panel. Antibody pairs from multiple manufacturers were tested for sensitivity with recombinant protein by titrating recombinant protein cocktails (5, 15.8, 50, 158, 500, 1580 and 5000 pg/mL) to produce a standard RFU/protein curve. (B) Antibody pairs were tested for specificity by spiking 1000 pg/mL protein standards for each antibody on the panel. Antibody pairs were then evaluated for cross reactivity within the panel. Antibodies were considered specific when the antibody pair had an SNR >10. (PDF 2103 kb) [file 40425_2017_293_MOESM4_ESM.pdf]

Additional file 5 (Figure S7)

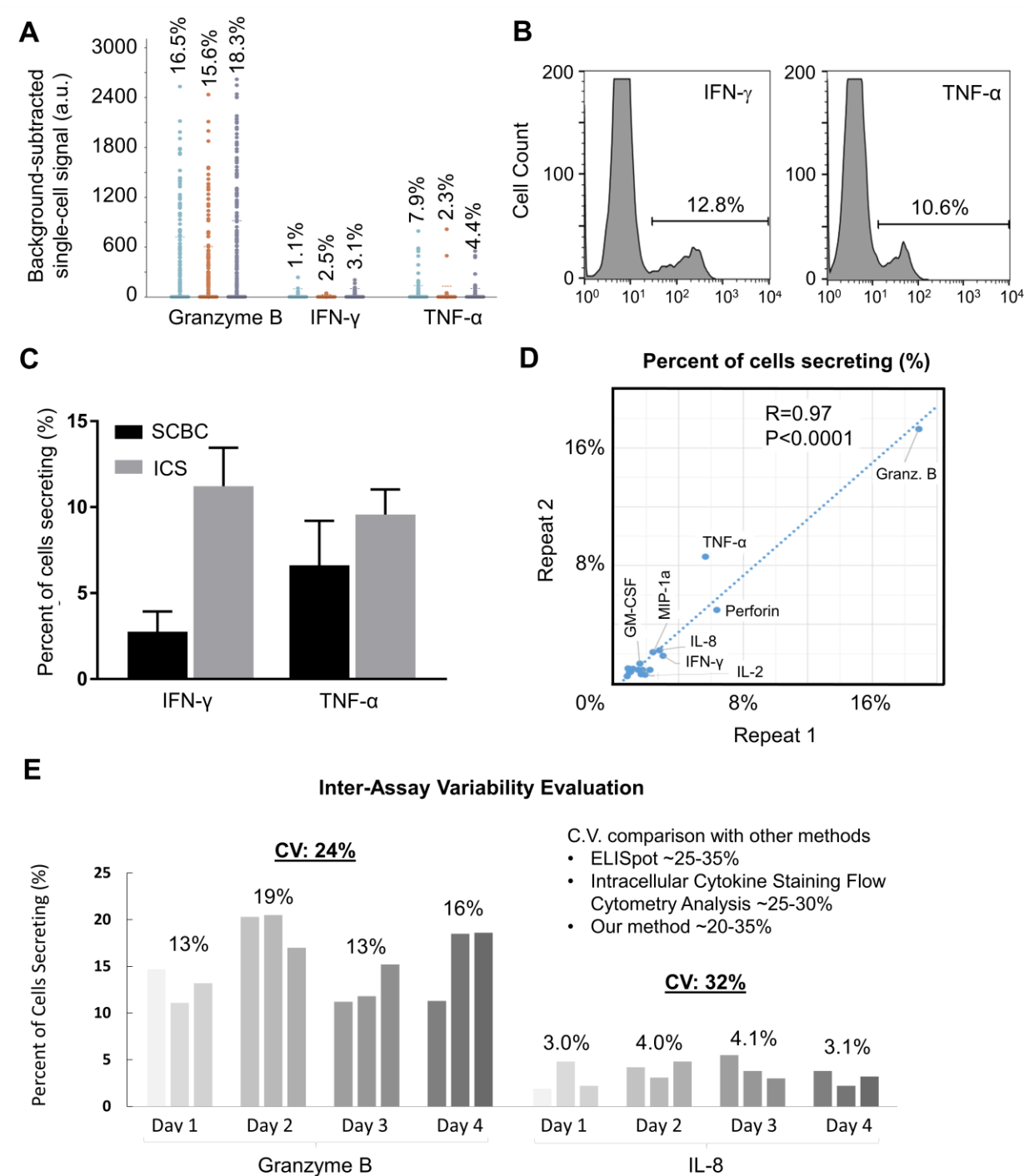

Supplement: Supplementary file 5 — Validation of the 16-plex cytokine panel on the SCBC platform. (A) A representative signal distribution of Granzyme B, IFN-γ and TNF-α from single CD8 T cells at the SCBC platform. (B) A representative ICS data of IFN-γ and TNF-α secreting CD8 T cells. (C) A pooled comparison data of IFN-γ and TNF-α secreting CD8 T cells between SCBC and ICS. (D) The correlation of 16 protein secretion levels between single-cell averages from two independent experiments (x, y axes: % of cytokine-secreting single CD8 T cells). (E) A representative scatter plots of Granzyme B and IL-8 from individual experiments. (PDF 3044 kb) [file 40425_2017_293_MOESM5_ESM.pdf]

Additional file 6 (Figure S2)

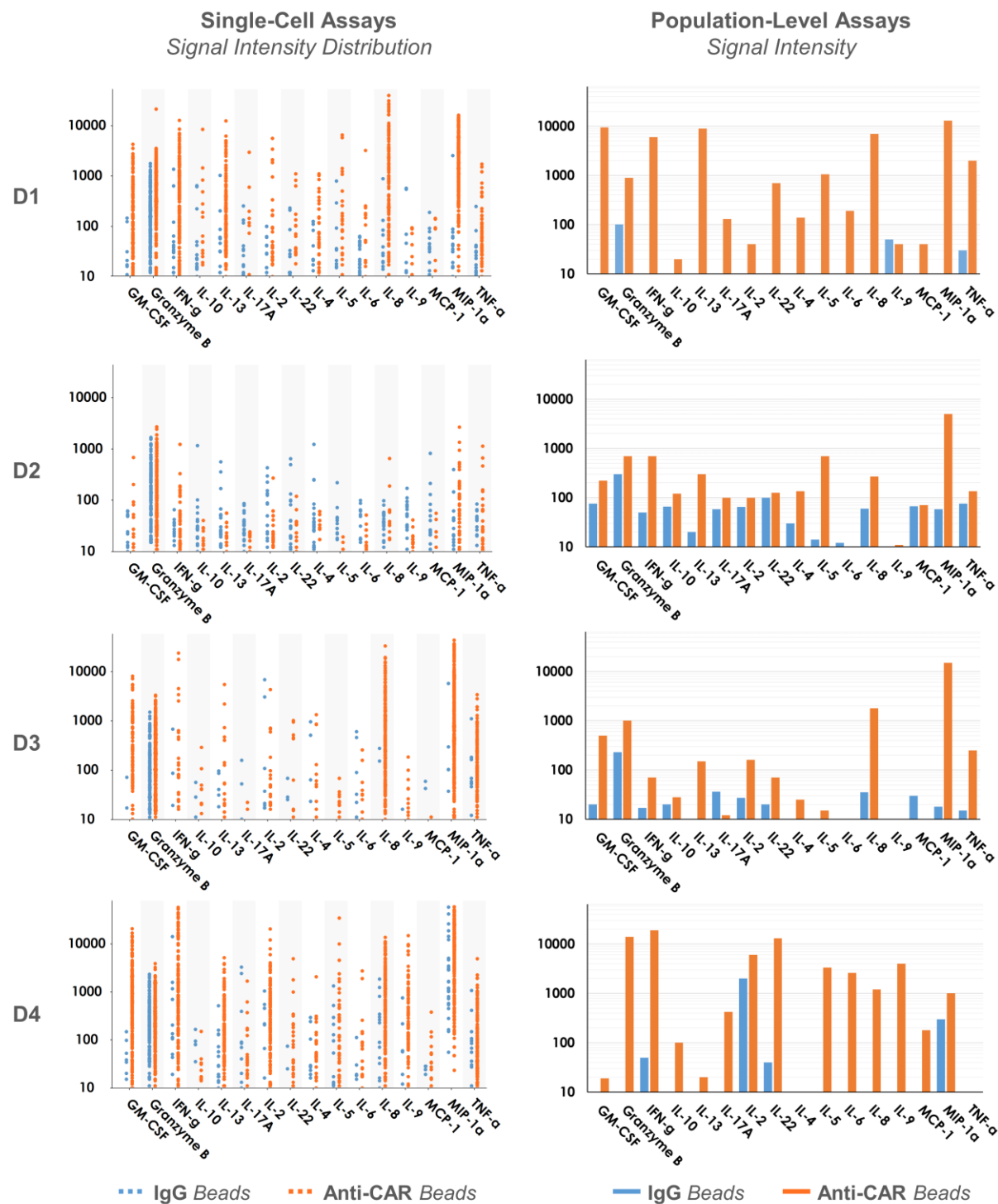

Supplement: Supplementary file 6 — The level of cytokine secretion from single cells and populations upon anti-CAR bead stimulation of CD19 CAR-T cells. At both the single-cell level and bulk-level, an overall increase in the intensity of effector and stimulatory cytokine secretions was observed with anti-CAR bead stimulation (orange) compared to control IgG bead stimulation (blue). While bulk-level measurements only show an average intensity per cytokine of the entire cell sample, single-cell level measurements present a full distribution of cell-by-cell secretion intensities. Levels of upregulation are consistent between the bulk-level measurement and single-cell level measurement across donors, with donor 2 having very small increases compared to the other three donors at both levels. (PDF 2103 kb) [file 40425_2017_293_MOESM6_ESM.pdf]

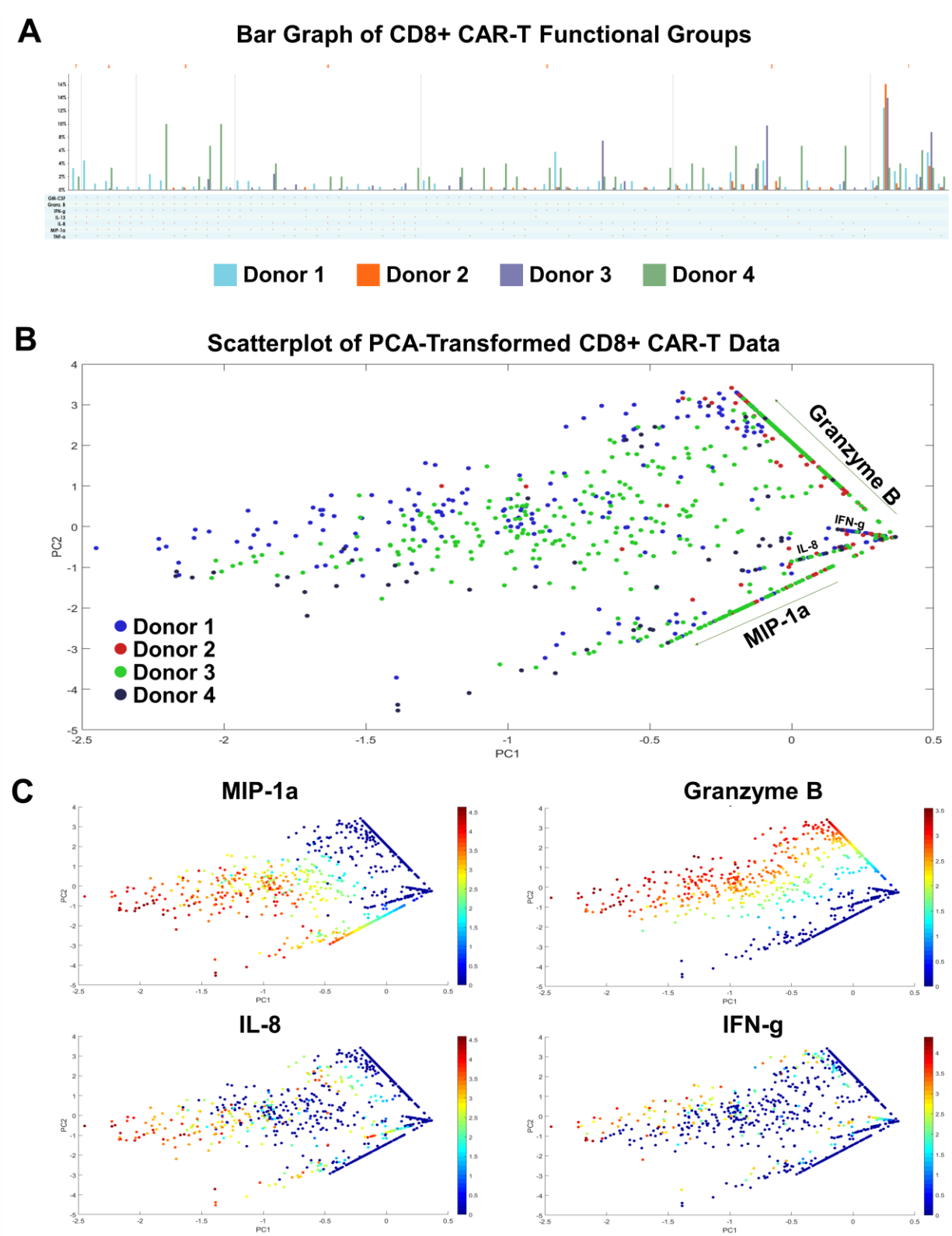

Supplement: Supplementary file 7 — Higher dimensional data is difficult to visualize concisely. (A) In this standard bar graph visualization of functional groups secreted by CD4+ CAR-T cells of four donors, it is cumbersome to see which are the major functional groups being secreted by each donor, and what are the biggest fold differences across donors. (B-C) Reducing the dimensionality of the dataset is a different approach to more effective and understandable visualizations. In this figure, PCA is applied to the 4-donor CAR-T secretion dataset. Each cell’s secretions (signal intensity of each cytokine) are log transformed prior to dimensionality reduction. (B) is color-coded by donor, while (C) is color-coded by some of the individual cytokines. The combination of these graphs reveals some information, such as the low overall polyfunctionality of donor 2, and the high Granzyme B+MIP-1a+ polyfunctionality of Donor 4. However, more detailed information about upregulated and/or distinct polyfunctional subsets is less clear. (PDF 2103 kb) [file 40425_2017_293_MOESM7_ESM.pdf]

Additional file 8 (Figure S5)

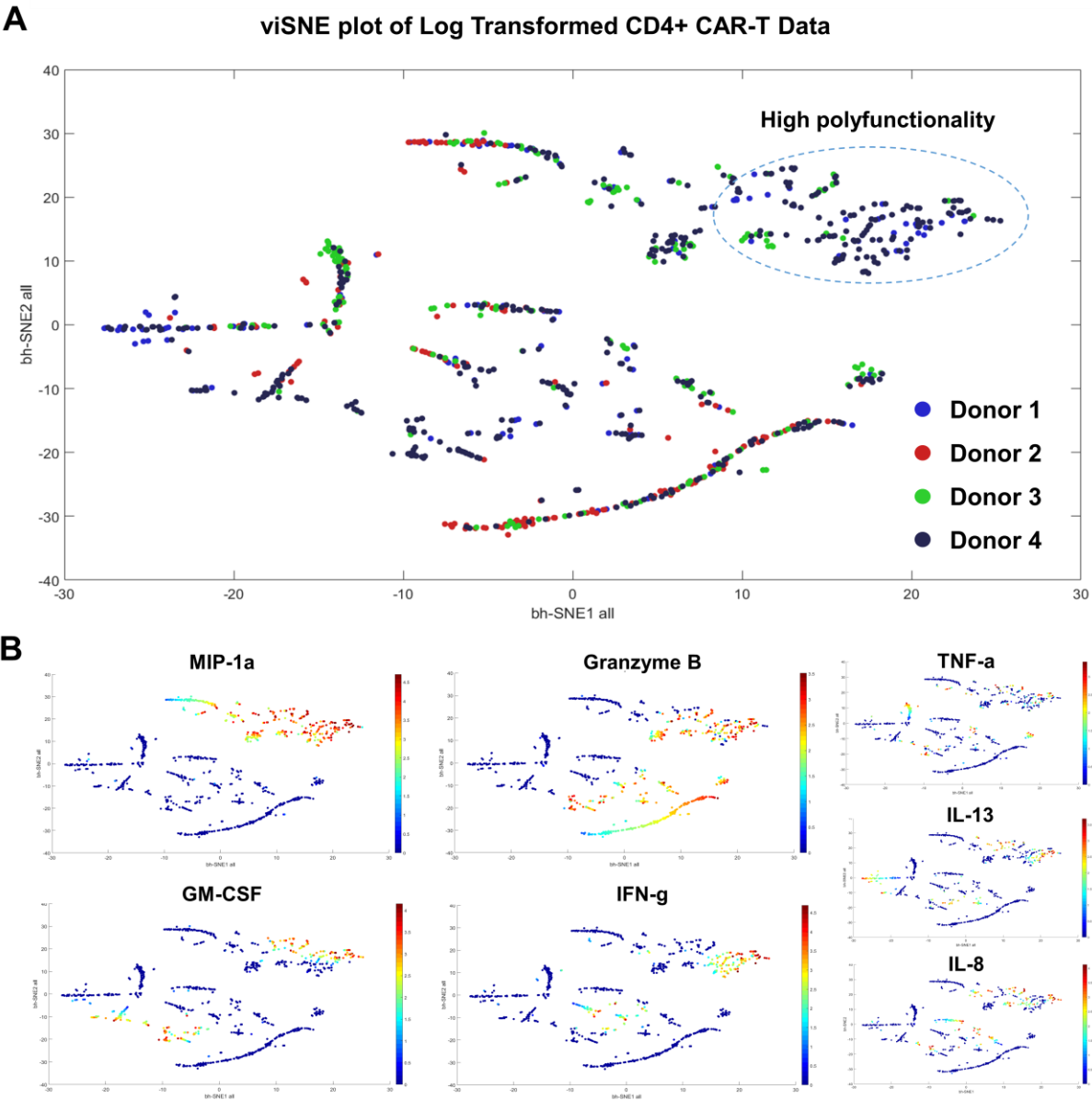

Supplement: Supplementary file 8 — viSNE visualization of CD4+ CAR-T data. viSNE is a visualization tool designed to map high-dimensional flow cytometry data onto two dimensions, while preserving the overall structure of the data. Similar to PCA, color can be used as a third dimension in the resulting visualization. In this figure, color is used to indicate (A) the donor sample of each single CD4+ CAR-T cell or (B) the intensity of individual cytokine secretions of each CD4+ CAR-T cell. Unlike PCA, which is a linear transformation, the benefit of visNE is its ability to preserve non-linear relationships across the data. One can infer that a subset of cells in each donor secrete only Granzyme B, that primarily donors 3 and 4 have cells secreting only TNF-a, and that donors 1 and 4 both have unique subsets of highly polyfunctional, Granzyme B + MIP-1a + IFN-g + secreting cells. However, additional donor differences and specific information about functional groups is fairly limited. A viSNE transformation of the CD8+ CAR-T data gives a similar graph. (PDF 2866 kb) [file 40425_2017_293_MOESM8_ESM.pdf]
